# The Early Evolution of Tudor Genes in Holozoa and How Their Distribution Was Influenced by Life History Traits in Metazoa

**DOI:** 10.1093/gbe/evaf051

**Published:** 2025-06-09

**Authors:** Giovanni Piccinini, Umberto Valdrè, Liliana Milani

**Affiliations:** Department of Biological, Geological, and Environmental Sciences, University of Bologna, Bologna, Italy; Department of Biological, Geological, and Environmental Sciences, University of Bologna, Bologna, Italy; Department of Biological, Geological, and Environmental Sciences, University of Bologna, Bologna, Italy

**Keywords:** gene family evolution, piRNA pathway, Piwi, parasitism, parthenogenesis, Ichthyosporea

## Abstract

Early metazoan evolution was characterized by the expansion of multiple gene families, such as the Tudor family, involved in novel multicellularity-related functions. In eukaryotes, Tudor genes (i.e. genes including at least one Tudor domain) are numerous, heterogeneous, and mostly associated with gene expression regulation. However, they underwent an animal-specific expansion, with novel elements almost exclusively involved in retrotransposon regulation through Piwi-interacting RNAs, as spatiotemporal regulators of the key-element Piwi, another previously considered animal-specific gene. Here, we used online-available proteomes covering 25 major taxonomic groups to characterize the Tudor gene family at a holozoan-wide level, confirming the apomorphic metazoan expansion of Piwi-interacting RNA-related Tudor genes. However, we also annotated elements of the Piwi-interacting RNA pathway (Tudor and Piwi genes) in Ichthyosporea species, suggesting that elements of the Piwi-interacting RNA pathway were already present in the holozoan common ancestors. We observed an outstanding variability (34-fold) of Tudor gene number between and within metazoan phyla that could be associated with convergent genomic and phenotypic evolutions: expansions were usually sided by whole-genome duplications and/or life history traits such as parthenogenesis; reductions were mostly associated to overall phenotypic and genomic simplifications, like in almost all considered endoparasites. Lastly, we phylogenetically tested, and mostly (but not completely) confirmed, a previously proposed model for the evolution of the Tudor domain secondary structures.

**Significance**

Similarly to other multicellularity-related gene families, many novel Tudor genes (i.e. genes including at least one Tudor domain) evolved in animals, with functions mostly associated to an animal-specific pathway of genomic defense against mobile elements. However, we observed that some elements of such pathway were already present in Ichthyosporea, early diverging unicellular Holozoa. Moreover, we observed an astonishing variability of the number of Tudor genes across and within animal phyla that can be associated to genomic dynamics, lifestyle, and reproduction modalities characterizing the different taxonomic groups. Collectively, our analysis of early evolution and within Metazoa diversification of the multifaceted Tudor protein family underlines the importance of considering lineage-specific evolutionary dynamics and provides valuable considerations about the co-option of genetic elements at the root of animal multicellularity.

## Introduction

The Tudor domain is a protein–protein interaction domain that has been observed in multiple proteins shared across most eukaryotic species. Despite hints for remote homology that have been found with some prokaryotic domains (Gonzalez et al. 2017; Liao and Smirnov 2023), the Tudor domain is currently considered a eukaryotic synapomorphy. It is nearly 60 amino acids long and it folds into a β-barrel composed of five β-strands (Selenko et al. 2001). This core tertiary structure is shared with other domains, namely PWWP, MBT, Agenet, and Chromo domains, leading to group these functional units in a eukaryote-specific remote-homology domain superfamily named “Royal family” (Maurer-Stroh et al. 2003). All members of this family can bind methyl-lysines (in mono-, di-, or tri-methylated states), while some Tudor domains evolved also the ability to bind symmetrically and asymmetrically di-methylated arginines (sDMAs and aDMAs, respectively; Cotè and Richard 2005 ; Chen et al. 2011; Lu and Wang 2013; Botuyan and Mer 2016).

**Fig. 1.**
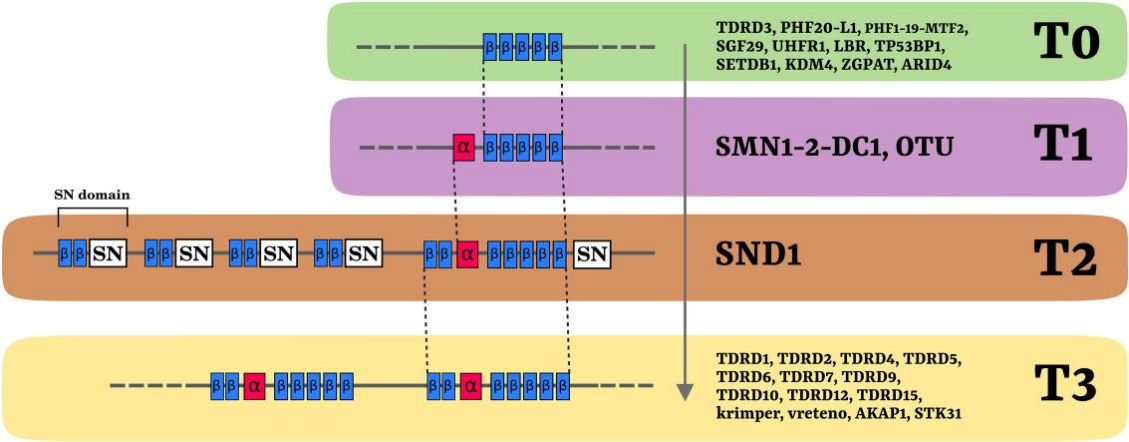
Structural division and evolutionary hypothesis of Tudor domains. The Tudor domains were previously grouped in four sets: T0 domains are constituted by five β-strands and are present usually in single or double copies in genes mostly involved in transcriptional regulation; T1 domains are present in fewer genes and are characterized by an additional α-helix N-t to the β-barrel core; T2 + T3 domains present two additional N-t β-strands and are present in the eukaryote-wide SND1 and in proteins of the animal-specific family expansion (present from single to multiple copies within the same sequence). Jin et al. (2009), who formalized the division, also proposed that the N-t structures were progressively acquired in a stepwise model from an ancestral T0 condition and that the animal T3 expansion was characterized by the co-option of the SND1 T2 domain. See Table 1 for gene-specific functions and literature.

The Tudor domain, often in combination with multiple other domains, is present in a largely diverse set of protein-coding genes (Tudor domain-containing genes, Tudor genes from now on) whose functions are usually conserved among species. The product of these genes (that we will refer to as Tudor proteins) is involved in a great variety of molecular pathways (Siomi et al. 2010; Chen et al. 2011; Pek et al. 2012; Lu and Wang 2013; Botuyan and Mer 2016; Table 1), and their functional framework is associated to the different secondary structures of their Tudor domains that are classified into four groups (here called T0, T1, T2, and T3; Jin et al. 2009; Fig. 1).

**Table 1:** Nomenclature and function of known Tudor proteins

Protein name (*H. sapiens*)	Protein name (*D. melanogaster*)	Tudor domain	Co-occurrent domains	Functions	References (see Supplementary material)
TDRD3	TDRD3	Single T0	UBA	Recognizes methyl-arginines on histones and on the C-terminal domain of RNApol-II; positive regulation of gene expression; included in stress granules, in association with FMR, probably sharing translational repression functions	Linder et al. (2008); Yuan et al. (2021)
PHF1-19-MTF2	Polycomb-like	Single T0	PHD	Stimulates catalytic activity of Polycomb repressive complexes 1 and 2 that are histone silencers involved in transcriptional repression	Dong et al. (2020)
PHF20-20L1	MBD-R2	Double T0	…	Subunit of lysine acetyltransferase complex that acetylates histone H4 and stabilize the tumor suppression protein p53	Cui et al. (2012)
SGF29	SGF29	Tandem T0	…	Component of the SAGA complex, a positive regulator of gene expression	Bian et al. (2011)
UHRF1	…	Tandem T0	UBL, PHD, SRA, RING	Methyl-histone-binding protein that recruits DNMT1 to recently replicated hemi-methylated DNA to facilitate efficient remethylation; sensor of DNA interstrand crosslinks	Bostick et al. (2007); Liang et al. (2015)
LBR	LBR	Single T0	Tm, RS region, Globular region II	Transmembrane protein of the inner nuclear membrane proposed as chaperone-like docking platform for heterochromatin assembly; also involved in cholesterol biosynthetic pathway	Liokatis et al. (2012); Nikolakaki et al. (2017)
TP53BP1	…	Tandem T0	BRCT	Involved in double-strand DNA break repair through promoting nonhomologous end joining and inhibiting homologous recombination DNA repair	Bunting et al. (2010); Callen et al. (2013)
SETDB1	Eggless	Tandem T0	MBD, SET	Histone methyltransferase that tri-methylates K9 of histone H3, inducing transcriptional repression; regulator of tumor suppressor protein p53	Ayyanathan et al. (2003); Fei et al. (2015)
KDM4A-B-C	KDM4A-B-C	Hybrid tandem T0	JmjC, JmjN, PHD	Histone demethylase activity associated to transcriptional activation	Whetstine et al. (2006); Labbé et al. (2014)
ZGPAT	ZGPAT	Single T0	ZnF-CCCH	Transcriptional repressor through recruitment of the nucleosome remodeling and deacetylase complex	Li et al. (2009); Gui et al. (2012)
ARID4A-B	Hat-trick	Tandem T0	RBB1 N-t, Arid/Bright, CHROMO	Gene suppressor and epigenetic regulator	Gong et al. (2021)
SMN1-2-DC1	SMN-SPF30	Single T1	…	Components of the SMN (survival of motor neuron) complex of ribonucleoprotein assembly; binds spliceosomal Sm proteins, involved in spliceosomal small nuclear ribonucleoprotein assembly	Kolb et al. (2007); Chen et al. (2011)
OTUD4	OTU	Single T1	OTU	Deubiquitinating enzyme, RNA binding with suggested functions in translation regulation, germ cell division, and differentiation in *Drosophila*	Steinhauer and Kalfayan (1992); Mevissen et al. (2013); Das et al. (2019)
SND1	Tudor-SN	Single T2	SN	Positive regulator of gene expression; spliceosomal small nuclear ribonucleoprotein assembly; miRNA RISC-mediated RNA interference; component of stress granules; involved in piRNA pathway	Reviewed in Gutierrez-Beltran et al. (2016)
TDRD1	CG9684/CG9925	Multiple T3	ZnF-MYND	piRNA pathway; Ago3/Piwi-binding	Chen et al. (2011); Vagin et al. (2009); Ku and Lin (2014)
TDRD2	Papi	Single T3	KH	piRNA pathway, Ago3/Piwi-binding	Chen et al. (2011); Liu et al. (2010); Ku and Lin (2014)
TDRD4	Qin	Multiple T3	ZnF-RING	piRNA pathway; Aub/Ago3/Piwi-binding	Ku and Lin (2014)
TDRD5	Tejas	Single T3	Lotus	piRNA pathway; Aub-binding	Yabuta et al. (2011); Ku and Lin (2014)
TDRD6	Tudor	Multiple T3	…	piRNA pathway; Aub/Ago3/Piwi-binding	Chen et al. (2011); Ku and Lin (2014)
TDRD7	Tapas	Multiple T3	Lotus	piRNA pathway; Piwi-binding	Tanaka et al. (2011); Ku and Lin (2014)
STK31	…	Single T3	PK	piRNA pathway; Piwi-binding	Chen et al. (2011)
TDRD9	Spindle-E	Single T3	DEAD/DEADH, HELICc, HA2	piRNA pathway; Aub-binding	Vagin et al. (2009); Ku and Lin (2014)
TDRD10	…	Single T3	RRM	Unknown	…
TDRD12	Yb-SoYb-BoYb	Single T3	DEAD/DEADH	piRNA pathway; Ago3/Piwi-binding	Ku and Lin (2014)
TDRD15	…	Multiple T3	…	Unknown	…
AKAP1	…	Single T3	Tm, KH	Regulation of mitochondrial functions; binding of PKA regulatory subunits	Livigni et al. (2006)
…	Krimper	Single T3	ZnF-CCCH	piRNA pathway, Ago3-binding	Sato et al. (2015)
…	Vreteno	Multiple T3	…	piRNA pathway	Zamparini et al. (2011)

The extended list of references included within this table is in Supplementary material.

T0 domains fold into the canonical β-barrel core and are present in a wide range of proteins shared by most eukaryotic lineages (here called T0 genes/proteins). Most of them possess histone “reading” activities, spacing from gene expression regulation to cell cycle regulation, DNA repair and methylation, and heterochromatin formation (Table 1). While most of these proteins harbor a single Tudor domain, some have two, closely associated in their secondary and tertiary folding, forming the so-called Tandem Tudor domain (namely TP53BP1, SGF29, SETDB1, UHRF1, and KDM4A/B; Botuyan and Mer 2016). All T0 Tudor domains bind specifically to methylated lysines, except for TDRD3 that has sDMA-binding activities.

T1 domains display an additional N-terminal (N-t) α-helix to the canonical β-barrel and bind sDMAs (Chen et al. 2011; Fig. 1). These Tudor domains characterize the closely related eukaryotic proteins SMN1 and SMNDC1, which are components of the SMN complex, involved in the assembly, metabolism, and transport of different ribonucleoproteins, such as spliceosomal small nuclear ribonucleoproteins (Kolb et al. 2007; Table 1). This Tudor domain type is also present in orthologs of *Drosophila melanogaster* OTU protein, which is a deubiquitinase with roles in female germline determination and proliferation (Pauli et al. 1993; Glenn and Searles 2001; Table 1), and the closely related ALG13, which acts in N-glycosylation of polypeptide chains (Gao et al. 2005).

T2 and T3 domains show an additional N-t extension and characterize SND1 and the germline-related Tudor proteins. Here, two β-strands and one α-helix precede the β-barrel core, and they all display sDMA-binding activities (Fig. 1). SND1, which is the only Tudor protein included in the third group (T2 in the present study), is present in most eukaryotes and is involved in a great variety of molecular pathways of expression regulation, from RNA interference through RNA-induced silencing complex-mediated miRNA (Caudy et al. 2003) to splicing (Gao et al. 2012), and also in stress response and other functions (reviewed in Gutierrez-Beltran et al. 2016; Table 1).

T3 domains, on the other hand, are specific of the germline-related Tudor proteins (here referred to as T3 proteins, i.e. containing at least one T3 domain) consist in a conspicuous number of factors that often include multiple T3 domains. Homologs of such proteins can be found exclusively in Metazoa, and their functions appear closely associated and limited to the germline-specific Piwi-interacting RNA (piRNA) pathway of transposable element (TE) silencing (Liu et al. 2010; Siomi et al. 2010; Table 1). This pathway is an RNA-mediated pathway for the silencing of retrotransposons that has been observed exclusively in animals and whose functioning is crucial for the proper formation of the germline and of totipotent somatic stem cell lineages (Juliano et al. 2010, 2011; Alié et al. 2015 ; Fierro-Constaín et al. 2017).

The authors that formalized the above classification (Jin et al. 2009) also proposed that the functional specialization of the four Tudor domains was linked to the progressive acquisition of the N-t extensions. These acquisitions may have provided new intrinsic properties to the domains, which emerged as new molecular mechanisms (the binding of methylated arginines) and biological functions. According to this “stepwise” acquisition model:

- The T0 domain secondary structure is the ancestral state, which binds exclusively methylated lysines, as suggested by the fact that also other Royal family domains are composed of 5 β-strands only.
- The ancestor of SMN-related Tudor proteins (T1 proteins) acquired the α-helix extension (ALG13 and OTU were not considered by the authors), shifting the molecular functions to methylated arginine binding.
- The insertion of a T1 domain within a SN domain of SND1 explains the emergence of T2 domain architecture and allowed to acquire biological functions associated to the small RNA machinery. This is suggested by the fact that SND1 is composed of four complete SN domains and a Tudor domain inserted within the fifth SN domain, in C-terminal position to the two β-strands.
- Subsequently, this Tudor domain together with the two N-t β-strands was co-opted during the Metazoa-specific expansion in the evolution of germline-related Tudor genes (T3 genes), specifically specialized in the piRNA pathway through the concurrent evolution of Argonaute proteins with N-t RG motifs that are Piwi and its close homologs (Ago3 and Aub in *D. melanogaster*).

The last point has implications on the major novelty of multicellularity in animals. Indeed, germline-related Tudor proteins are part of the germline multipotency program (GMP), a set of genes that are involved in primordial germ cell specification and maintenance of long-term stem cell multipotency (Juliano et al. 2010). The emergence of multicellularity was sided by the evolution and/or the expansion of many gene families involved in cell lineage specification and tissue differentiation, including GMP genes. The presence of multiple Tudor domains in germline-related Tudor proteins suggests that they might act as docking platform for spatial organization of the components, forming typical germline granules. Specifically, the binding affinity of germline-related Tudor proteins with Piwi-like homologs suggests that they participate in the piRNA pathway as scaffold proteins (Juliano et al. 2011; Siomi et al. 2011; Lim and Kai 2015).

A certain degree of variability in the set of Tudor proteins among different lineages has been observed. For example, in well-studied organisms, such as *Drosophila* species and mammals, multiple lineage-specific Tudor genes have been observed, suggesting a flexibility in the composition of the Tudor gene set. Moreover, previous comparisons between teleosts and mammals have shown lineage-specific duplications and/or loss of T3 genes in fish that are also associated with higher rates of sequence evolution (Yi et al. 2014; Chen et al. 2017; Liu et al. 2022). The authors interpreted these events in terms of adaptive evolution acting on the piRNA pathway required for gametogenesis in such species, invoking TE dynamics and life history traits such as external fertilization to interpret the different evolutionary dynamics.

However, the Tudor family as a whole was rarely considered in evolutionary studies. Therefore, in the present study, we aimed to investigate the evolution of the Tudor domain and of the whole Tudor domain-containing gene family throughout the whole Metazoa clade and closely related unicellular Holozoa, using proteomic data from online available genomes. First, the enormous variability that we observed in the number of Tudor genes allowed us to make some considerations about the driving forces that might explain the dynamics of expansion and reduction of this gene family in the animal lineage. We, therefore, got insight on whether genomic features, piRNA pathway evolution, and specific life history traits might be driving forces in the evolution of the metazoan different Tudor protein sets.

Then, we tested the stepwise model of N-t extension acquisition in Tudor domains through phylogenetic tools, comparing expectations of the model (like monophyly of T1 + T2 + T3 domains or sister relationships between T2 and T3 domains) with observed relationships retrieved by profile-based and alignment-based tree inferences.

## Results

### Tudor Protein Homology Group Composition

We downloaded proteomes from online databases for a total of 111 holozoan species, comprehending 21 metazoan phyla and 4 major taxonomic groups of unicellular Holozoa (supplementary table S1, Supplementary Material online). We kept only the longest isoform for each gene, and we clustered homologous sequences with OrthoFinder v2.3.11 (Emms and Kelly 2019). Crossing the homology clusters (OrthoFinder's orthogroups or OGs), with domain annotation by InterProScan v5.45.80 (Jones et al. 2014), followed by within-cluster HMMER v3.2.1 iterations (Eddy 2011; see Materials and Methods for details), we identified 33 OGs comprising at least ten Tudor proteins of at least two species (therefore here named Tudor OGs). Of these, 21 contained Tudor proteins previously annotated and characterized in model species (we will refer to them as “annotated OGs” from now on), while 12 contained Tudor proteins that were never named in other works (“new OGs”: since they are not annotated, they are named with the OrthoFinder nomenclature). Of the latter, only four OGs included more than two phyla, suggesting that these Tudor proteins likely reflect lineage-specific evolutions.

The total number of Tudor proteins we identified was 3,323, most of which (2,266) ended up in the annotated OGs, 178 in the new OGs, while 279 were either not included in any OG considering our threshold for defining OGs (see Materials and Methods). Of the Tudor proteins included in the annotated OGs, more than one-third (900 proteins) ended up within the same homology group (OG164 referring to OrthoFinder nomenclature), which collected most Metazoa-specific T3 proteins: namely TDRD1-2-4-5-6-7-15, and AKAP1 (following *Homo sapiens* nomenclature). This “noisy” OG comprising multiple multi-Tudor proteins likely reflects a bricolage-like complex evolutionary pattern of Tudor domain acquisition that these proteins experienced during the metazoan radiation, leading to many cases of multiple occurrences within the same protein. To improve the resolution of subsequent analyses, we subsampled the OG164 in eight subclades with DISCO (Willson et al. 2022 ) that decomposes gene family trees in orthology groups based on a reference species tree.

### The Variability in Numbers of the Different Sets of Tudor Proteins Within Metazoa Is Associated to Different Genomic/Genetic Features

**Fig. 2.**
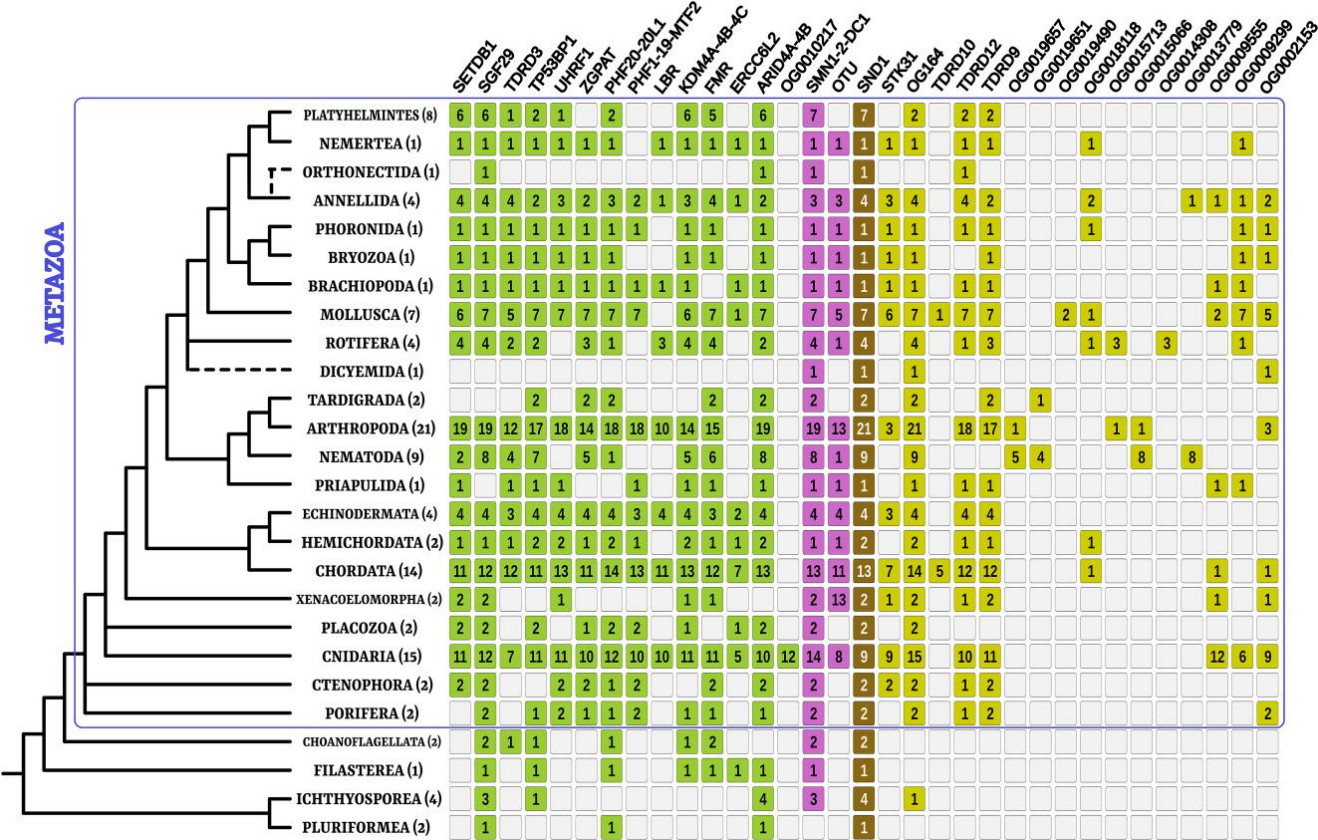
Phylum-specific patterns of presence/absence of Tudor OGs. Most T0 and T1 genes could be annotated in unicellular holozoan. Tudor OG annotation (vertebrate or *D. melanogaster* nomenclature) is depicted on the upper side; OrthoFinder nomenclature (e.g. OG0019657) is shown for the “new OGs” since they lack annotation. Relationships between clades are schematized on the left (mostly based on López-Escardó et al. 2019 and Laumer et al. 2019; inner spiralian relationships refer to Bleidorn 2019, with ambiguous phyla positioned with dotted line). The numbers in parenthesis following clade names refer to the number of species considered in the present study. Numbers within colored squares refer to the number of species comprising at least one Tudor-containing sequence in that OG. Colors refer to Tudor gene sets schematized in Fig. 1.

**Fig. 3.**
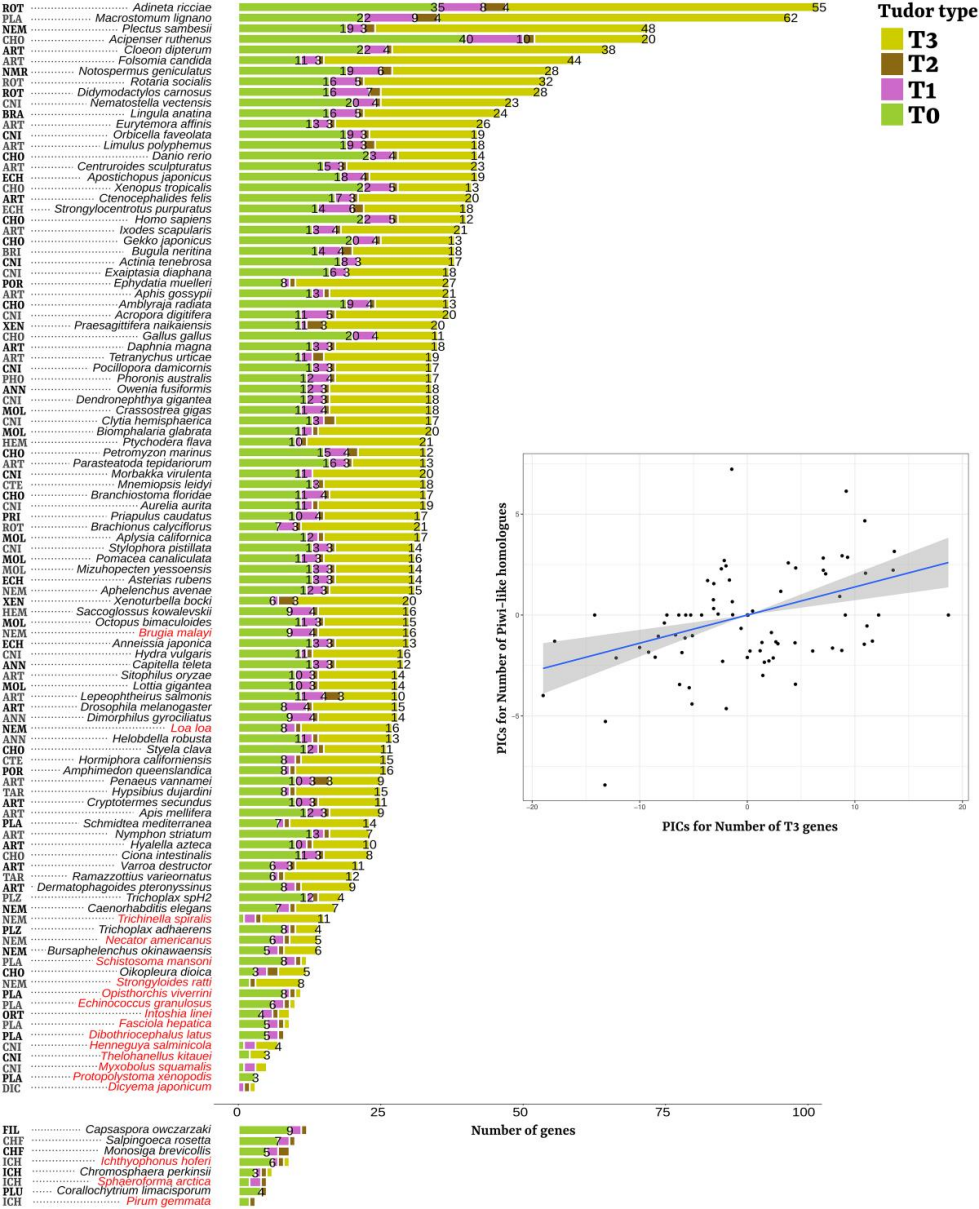
Barplot of Tudor gene numbers across species and correlation between number of T3 and Piwi homologs. Species are ordered based on the total number of Tudor genes we annotated. Unicellular holozoan is shown in a separate barplot on the bottom. There is a 34-fold variability in the total number of Tudor genes in the species of our dataset (endoparasites depicted in red). The number of genes (higher than 1) belonging to each Tudor set is depicted in the top of the respectively colored barplot. Nonmetazoa Holozoa is separated on the bottom right of the figure. On the left of each species, is an abbreviation of the first three letters of the belonging phylum (exceptions due to homonymy: NMR, Nemertea; PLZ, Placozoa; CHF, Choanoflagellata). On the right side of the figure, the linear regressions for PICs of the number of T3 genes and the number of Piwi-like homologs (*ρ* = 0.429; corrected *P* = 6.826 ∗ 10^−4^; see Materials and Methods). PIC values were calculated on the distributions after removing species below the 10th and over the 90th percentiles of the distribution of the total number of Tudor genes.

We could identify the great majority of annotated Tudor genes in almost all animal phyla, including early-branching clades like Porifera and Ctenophora, confirming the metazoan-wide distribution of the whole Tudor protein set (Fig. 2). Within Metazoa, we observed a conspicuous variation in the number of Tudor genes, even within the same phylum (Fig. 3). Moreover, the distributions of the four Tudor groups (T0, T1, T2, and T3) differed both in terms of absolute values and coefficient of variation. This, together with the functional differences explained in the Introduction, suggests that their evolutionary patterns should be considered separately. To better characterize the possible driving forces of the great variability in Tudor genes, we decided to explore possible correlation with a series of variables that consider the different functions of the four Tudor groups (Fig. 3; correlations were checked for phylogeny and tested after removing species below the 10th and over the 90th percentiles of the distribution of total number of Tudor genes: see Materials and Methods; all numerical values used for the correlations are present in supplementary table S2, Supplementary Material online, and a summary of all correlations is in supplementary table S3, Supplementary Material online). Because most of the T0 proteins are involved in epigenetic gene expression regulation and chromatin formation, we considered genome size (*C*-value) and gene density as simple proxies of genome complexity. T1 proteins are involved in posttranscriptional regulation, acting in the spliceosome complex (except for OTU), and thus, we considered the splicing index. Finally, T3 proteins (T2 genes were not considered in statistics since they comprise SND1 only) are involved in the piRNA pathway, and we investigated the correlation with the number of Piwi-like proteins (i.e. the OG comprehending Piwi, Ago3, and Aub sequences of *D. melanogaster*) and Ago-like homologs as a control (i.e. the OG comprehending Ago1 and Ago2 of *D. melanogaster*, involved in non-piRNA silencing mechanisms). All correlations were corrected for phylogenesis.

- The number of T0 proteins was mildly correlated with the total number of genes, *C*-value, and gene density, but significance was not kept after correcting for false discovery rate (supplementary table S3, Supplementary Material online). The same is true for the number of T1 proteins that was weakly correlated with gene density before correction for multiple tests.
- Only the number of T3 proteins positively correlated with the total number of genes (*ρ* = 0.545, corrected *P* = 3.027 ∗ 10^−6^). Moreover, only T3 protein number significantly correlated with the number of Piwi-like genes: *ρ* = 0.429, corrected *P* = 6.826 ∗ 10^−4^).
- No structural Tudor group correlated with the number of Ago-like genes.

### The Evolutionary History of the Tudor Domain During the Holozoan Diversification

To explore the evolutionary history of the Tudor domains and to test the “stepwise” model of evolution as proposed by Jin et al. (2009), we considered only domain sequences from Tudor OGs for which the iterative HMMER annotation was performed. We extracted each occurrence of a Tudor domain within each protein included in the Tudor OGs (see Materials and Methods for domain extraction details), and we followed two different phylogenetic approaches.

**Fig. 4.**
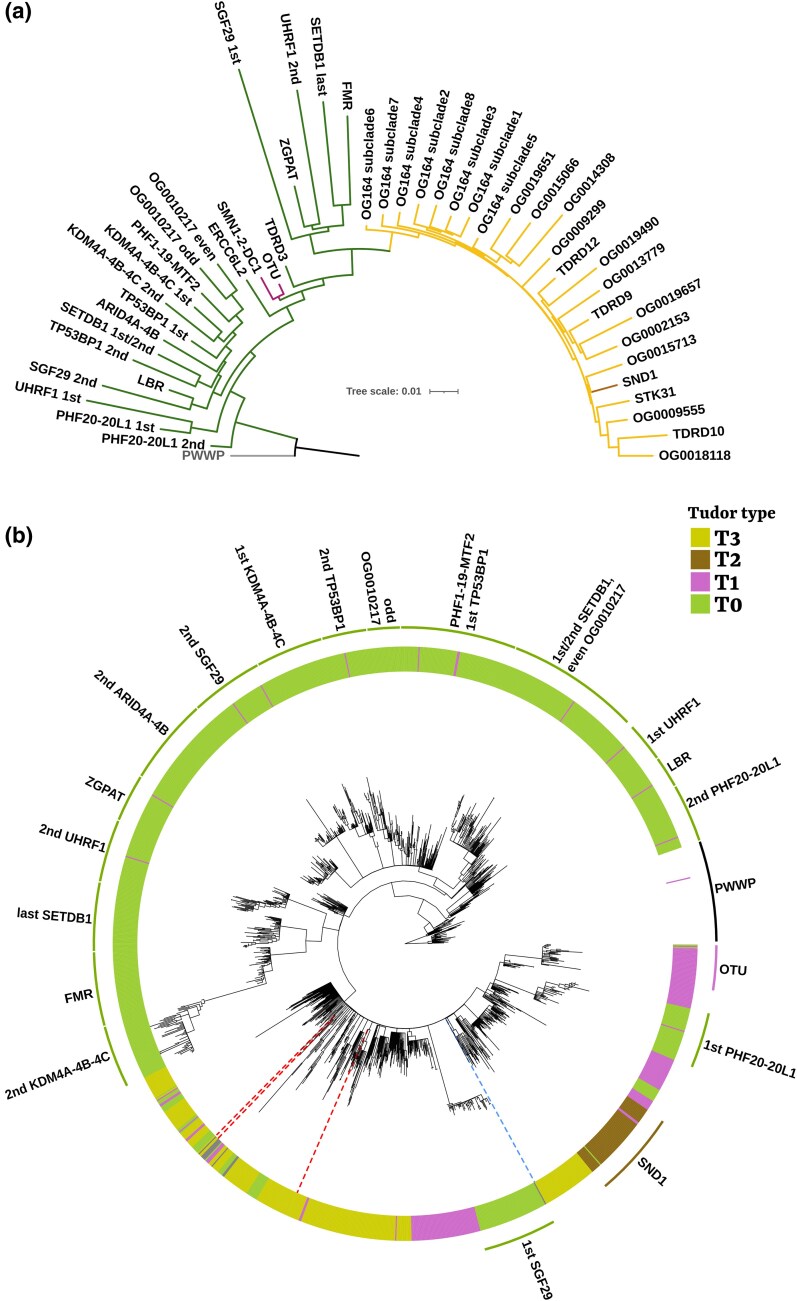
Phylogenetic reconstructions of the relationships between Tudor domains belonging to different OGs. Trees are rooted with PWWP domains. a) NJ tree of the alignment profiles of the Tudor domains. The tree was inferred (pHMM-Tree) based on the distance matrix between profiles built on alignments of Tudor domains belonging to the same OG. Branches are colored based on the Tudor gene set. b) ML tree of the Tudor domain alignment. A 50% subsample of filtered Tudor domains of all OGs (with the exclusion of the noisy OG164; see Materials and Methods) were aligned and ten ML tree replicates were inferred (IQTREE; three failed topology tests). The topology depicted in the figure is that of the replicate with the highest likelihood (others are shown as supplementary figs. S1 to S6, Supplementary Material online). UFB supports (1,000 replicates) were collapsed at 95%. Tudor types are shown in the circle surrounding the tree (together with OG nomenclatures for monophyletic groups). Blue dotted line: *I. hoferi* T3 sequence; red dotted lines: *C. perkinsii* T3 sequences.

First, we built a neighbor-joining (NJ) tree based on HMMER profile distances (Fig. 4a). For each Tudor OG, we built a profile for each occurrence of a Tudor domain (for instance, two separate profiles were built for the alignment of the first and second Tudor domains of TP53BP1) and inferred a NJ tree with pHMM-Tree (Huo et al. 2017) based on a profile distance matrix obtained with the PRC algorithm.

Second, we ran ten replicates of IQ-TREE to infer maximum likelihood (ML) phylogenetic tree topologies (each with 1000 UltraFast Bootstrap [UFB] iterations for support) on a subsample of Tudor domains: comprehending all “annotated” and “new” OGs, except for OG164 (see Materials and Methods). The ten replicate topologies were evaluated with the topology tests implemented by IQ-TREE, and three were significantly excluded by at least two methods (Fig. 4b represents one of the seven replicates; for others, see supplementary figs. S1 to S6, Supplementary Material online). Considering the enormous tree space that the software needed to explore, we decided to compare the different replicate topologies by stating how many times a discussed relationship was retrieved, rather than discuss a single consensus tree.

The NJ tree topology inferred is mostly concordant with the stepwise model of Tudor evolution (Fig. 4a; Jin et al. 2009). The T0 domain secondary structure is paraphyletic, with both T1, T2, and T3 domains nested within T0 clades, suggesting that the T0 type was indeed the ancestral Tudor state. The same pattern was retrieved also for all seven ML replicates (Fig. 4b), and indeed ancestral state reconstruction (ASR) analyses inferred the T0 domain as the ancestral Tudor state with ∼1 probability for all the considered replicates (see Materials and Methods). The second T0 domain of PHF20-20L1 proteins (involved in histone acetylation) shows the shortest distance from the PWWP outgroup in the NJ tree and appears in a sister relationship with all other Tudor domains in the NJ tree. Despite being often paraphyletic, this was observed also in four out of seven ML replicates.

As regards the evolutionary emergence of subsequent N-t extensions, in the NJ tree, both T1 and T2 + T3 domains form monophyletic groups. However, they are separated by a couple of nodes that include T0 domains, suggesting a single acquisition of the N-t α-helix followed by subsequent losses, or its independent evolution in the T1 and T2 + T3 groups. In the ML trees, despite retrieving T2 + T3 domains as monophyletic in five out of seven replicates (except for some not consistent single T0 domain sometimes nested within T2 domains), T1 domains were most of the time para- or polyphyletic. In most replicates (four out of seven), domains belonging to OTU proteins were either in sister relationship or included within the T2 + T3 domain clade, and domains belonging to SMN1-2-DC1 and TDRD3 were in a sister relationship with T2 and OTU ones. In the ASR analyses, however, the transition from T1 to T2 domain had the lowest probability for nearly all replicates (supplementary table S4, Supplementary Material online).

Regarding inner relationships of the T2 + T3 domain clade, the T2 domains belonging to SND1 proteins were never retrieved in a sister relationship with all T3 domains (neither in the NJ tree nor in the seven ML trees). However, the branch lengths of the internal nodes that define the different T2-T3 domain clusters were usually very short, and deep nodes had lower support with respect to the rest of the tree, hinting toward a shorter divergence time among their common ancestors, possibly impeding accurate phylogenetic reconstruction. Indeed, by collapsing nodes with UFB support lower than 95 (as shown in Fig. 4b), T1 + T2 + T3 domains are still included in a monophyletic clade, but their relative relationships are not solved.

### piRNA-Related Elements Were Retrieved in Two Ichthyosporea Species

In most unicellular Holozoa we observed a discrete number of T0 and T1 genes, we could annotate SND1 (the only T2 gene), but barely no T3 gene was retrieved (Fierro-Constaín et al. 2017). Two notable exceptions were in the Ichthyosporea species *Ichthyophonus hoferi* (additional T3 gene belonging to OG164) and *Chromosphaera perkinsii* (a T3 gene belonging to a small OG comprising some Tardigrada sequences). In a recent work, proteins containing T3 domains (almost always coupled to Development and Cell Death [DCD] domains) were annotated in five Choanoflagellata belonging to the two major clades of loricate (Acanthoecida) and non-loricate (Craspedida) species (Bell et al. 2020). No sequence similarity could be retrieved between the five choanoflagellate T3 + DCD proteins from the literature and the two ichthyosporean T3 proteins of the present study. Moreover, no Tudor domain nor protein homologous to those annotated by Bell et al. could be annotated in none of the Choanoflagellata included in the present analysis (supplementary table S5, Supplementary Material online).

To get insight on whether the two ichthyosporean T3 genes we retrieved are the results of horizontal gene transfers (HGTs) from animals (most Ichthyosporea are endoparasites of animals; in our dataset, only *C. perkinsii* is free-living to present knowledge; Grau-Bové et al. 2017), we included their T3 domains in our Tudor domain ML tree topology. We ran ten ML replicates forcing the previously retrieved topology (see previous Results subsection) and allowing for the ichthyosporean T3 domains to be freely placed in the tree. *Ichthyophonus hoferi* T3 domain was included into a polytomic clade (after UFB collapse) comprising domains belonging to SND1, STK3, and OG0009555, while the three T3 domains of *C. perkinsii* ended up scattered among metazoan T3 domain noisy relationships (dotted colored branches in Fig. 4b).

To test different a priori topologies and compare their likelihood, we first built ten ML tree replicates of an alignment of T3 domains belonging to OG164 subclades (including *I. hoferi* T3 domain) and T2 domains of SND1 proteins (as outgroup). We compared the likelihood of the topologies with ten replicates for which we forced *I. hoferi* in a sister relationship with all other OG164 domains. These latter topologies did not have significantly lower likelihoods (for none of the IQTREE topology tests; Minh et al. 2020); therefore, *I. hoferi* T3 domain as sister to all T3 domains belonging to OG164 stands as a plausible hypothesis (trees in Supplementary material).

Interestingly, *I. hoferi* and *C. perkinsii* were the only two holozoan species for which we also identified sequences included in the homology cluster of Piwi proteins (including Piwi, Ago3, and Aub of *D. melanogaster*). The two ichthyosporean Piwi-like protein sequences were characterized by the precise domain architecture found in metazoans (Piwi + PAZ domains), that is, however, also shared by close homologs of Ago1 and Ago2, that are not involved in the piRNA pathway. We, therefore, built HMM profiles for both Piwi and PAZ domains of both OGs (Piwi-like and Ago-like; excluding Ichthyosporea sequences) and found that *C. perkinsii* and *I. hoferi* Piwi and PAZ domain sequences better aligned with the profiles built on the Piwi-like OG, suggesting that they could indeed be putative holozoan homologs of the Piwi-related proteins. Coherently, both ichthyosporean Piwi-like proteins (especially *I. hoferi*) shared the presence of characteristic N-t RG conserved motifs (supplementary fig. S7, Supplementary Material online)

**Fig. 5.**
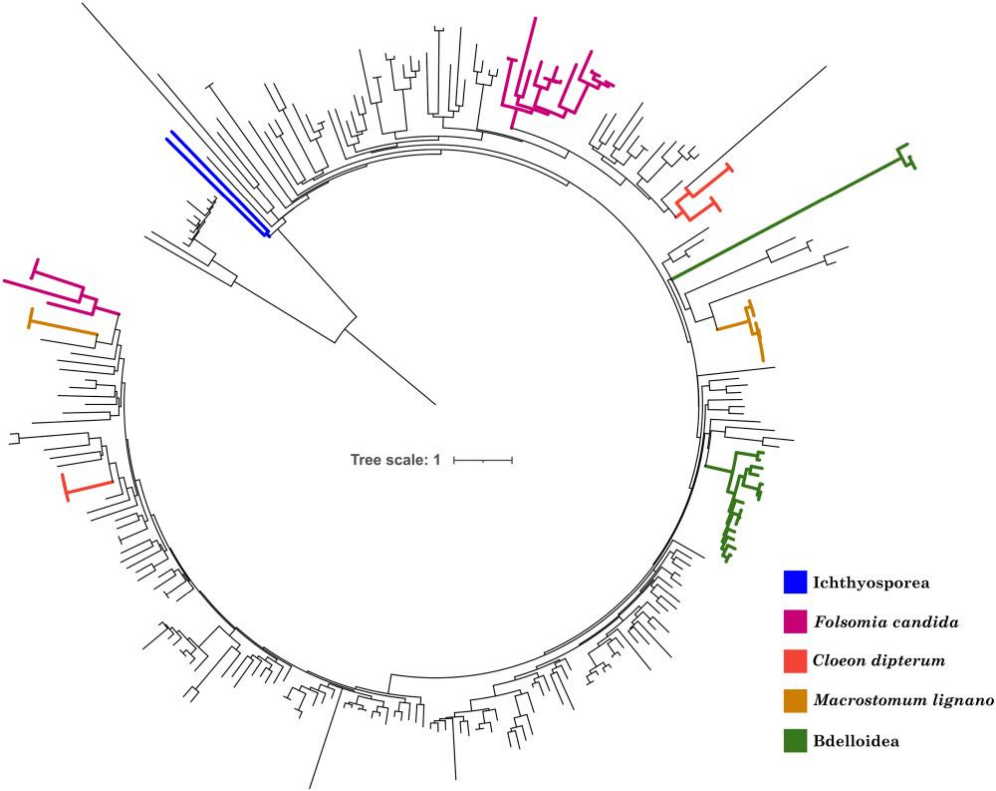
ML tree topology of Piwi + PAZ domains of Piwi-like homologs in Holozoa. The tree was inferred from the alignment of concatenated and partitioned Piwi and PAZ domains of sequences belonging to the Piwi-like OG. The topology depicted refers to the highest likelihood IQ-TREE replicate (of ten replicates) inferred forcing ichthyosporean sequences in a sister relationship with metazoan ones (for inferences with no constraint or other topological constraints, at least one replicate failed topology tests; see Results). The tree is rooted with domains belonging to Ago-like sequences.

To better investigate the phylogenetic relationships of these proteins, we built ten replicates of ML phylogenetic trees based on partitioned alignments of Piwi and PAZ domains only. A subsample of sequences belonging to the Ago-like OG was used as outgroup (one sequence for each phylum). Sequences of *C. perkinsii* and *I. hoferi* clustered outside the Ago-like outgroups, confirming their higher similarity with Piwi-like metazoan sequences with an independent method. Nonetheless, in none of the ten ML replicates, *C. perkinsii* and *I. hoferi* were in a sister relationship with Metazoa, but they were rather nested within early diverging small metazoan clades. We then tested the likelihoods of a priori topologies against the free inferences: we tested ten topologies forcing the two ichthyosporean species in a sister relationship with animals, ten topologies forcing only *C. perkinsii*, and ten topologies forcing only *I. hoferi*. None of the 40 topologies was significantly better than the others with the approximately unbiased test (Shimodaira 2002), but the only case where all ten replicates passed all topology tests implemented by IQTREE (therefore them not being statistically worse than the free-inference topologies) was when forcing both Ichthyosporea Piwi + PAZ domains in a sister relationship to all animal ones (Fig. 5 depicts the Ichthyosporea-sister replicate with highest likelihood; all trees in Supplementary material).

## Discussion

### Reductions in the Number of Tudor Genes is (Often) Associated With Endoparasitism and Expansions With WGDs

Interestingly, nearly all dataset species with a severely reduced number of Tudor genes were endoparasites (most of these species did not drive the statistics since we removed the first and last deciles before correlating). For seven out of these eight independent evolutions of endoparasitism in our dataset (14 out of 16 endoparasite species; data on parasitism occurrences from the survey of Weinstein and Kuris 2016; supplementary table S6, Supplementary Material online), we could find a common pattern of strong reduction of Tudor genes (Fig. 3; supplementary table S2, Supplementary Material online).

Parasitism has been often associated in a causal manner to a reduction of phenotypic/genotypic complexity (Tsai et al. 2013; Zarowiecki and Berriman 2015). Indeed, we observed mere global gene content reduction, together with high gene density and small genome size, for many extreme parasites of our dataset: the phyla Orthonectida and Dicyemida (represented by one species each), the class Myxozoa (three species of highly derived parasitic cnidarian), and Neodermata (Platyhelminthes). This was associated especially to extreme reduction of T0 genes (five or less genes, i.e. lower than half of the overall median), with numbers conspicuously lower than their free-living close relatives, and even lower than some unicellular holozoan clades (Fig. 3).

Also, in Nematoda, we observed lower total number of genes for parasitic species compared to the free-living species *Caenorhabditis elegans*, *Aphelenchus avenae*, *Bursaphelenchus okinawaensis*, and *Plectus sambesii*. However, in these cases, the variability of the Tudor gene sets probably followed lineage-specific evolutionary pathways. Indeed, data from independent occurrences of parasitism evolution in Nematoda revealed how genome dynamics are highly variable and not straightforwardly associated with lifestyle strategies (Blaxter and Koutsovoulos 2015; Viney 2018). Thus, the phenotypic, genomic, and lifestyle evolutionary history of Nematoda appears extremely convoluted, and no unifying consideration can be made to explain their extreme variability.

For many species that showed Tudor gene number inflations (Fig. 3), on the other hand, we could identify lineage-specific whole-genome duplication (WGD) events that might explain the component of the Tudor set made by regulatory T0 genes, since regulatory genes and genes involved in protein–protein interactions are thought to be more often retained by mechanisms of dosage balance after WGDs (Freeling and Thomas 2006; Sémon and Wolfe 2007 ; Jiang et al. 2013): the free-living flatworm *Macrostomum lignano* (recent species-specific WGD; Zadesenets et al. 2017), three species of bdelloid rotifers (ancient tetraploidy in Bdelloidea; Hur et al. 2009), and *Acipenser ruthenus* (Acipenseriformes-specific WGD with high degrees of both structural and functional tetraploidy at present times; Cheng et al. 2019; Du et al. 2020). Moreover, usually the number of T0 genes is almost perfectly doubled compared to the closest relatives: in Bdelloidea rotifers with respect to the monogonont *Brachionus calyciflorus* (16 against 7; for *Adineta ricciae* enormous inflation, see Nowell et al. 2018) and in *A. ruthenus* with respect to other Craniata (40 T0 genes against a Craniata median of 21).

### The Evolutionary Patterns of T3 Genes Are Strongly Associated to Piwi Proteins and Reproductive Strategies

We found a strong correlation exclusively between the number of T3 genes and of Piwi homologs (Piwi, Ago3, and Aub; following *D. melanogaster* nomenclature). T3 proteins are almost exclusively associated with the piRNA pathway of retrotransposon silencing in the germline (Jin et al. 2009), and the correlation between these two gene groups reflects their role in the same molecular pathway. We could not find any correlation with Ago-related genes, confirming how the strict relationship with Piwi-like proteins is specific and limited to the piRNA pathway, rather than to generic Piwi domain-containing proteins.

In most of the species with an overall low number of Tudor genes (e.g. endoparasites and the two free-living species *Oikopleura dioica* and *Trichoplax adhaerens*), the reduction was mostly driven by reduction in the number of T3 genes, flanked by a reduction in the number of Piwi-like homologs. This was not sided by a similar trend for the Ago-like homologs (coherently with the exclusive and positive correlation between numbers of T3 and Piwi-like genes; Fig. 3). For instance, endoparasitic flatworms lost all Piwi homologs, as previously observed in other works (Tsai et al. 2013; Fontenla et al. 2021), sided by the complete loss of the piRNA pathway and most of its associated genes (Skinner et al. 2014; Fontenla et al. 2021), and T3 gene group reduction (median of 2 against an overall metazoan median of 16; T0 and T1 gene groups had a median of 9.5 against an overall metazoan median of 11 for T0 genes; median of 2 against 3 for T1 genes).

Also, the nematodes *Brugia malayi* and *Loa loa* did not have Piwi copies and, interestingly, they lost almost all classic piRNA-associated T3 genes: the T3 genes present were sequences included in nematode-specific OGs or in nonannotated OGs shared with few other phyla (supplementary fig. S8, Supplementary Material online). However, we could observe the same loss of canonical T3 genes in other Nematoda who also lost Piwi (endoparasites *Trichinella spiralis* and *Strongyloides ratti* and the free-living *A. avenae*) and also in species that did not lose it, like *Necator americanus* and the free-living *B. okinawaensis* and *C. elegans* (supplementary fig. S8, Supplementary Material online), suggesting that in Nematoda, the Tudor gene loss is a common pattern, despite lifestyle and *piwi*-related dynamics.

Coherently, species with a conspicuously higher number of Tudor genes mostly coincided with those with the highest number of Piwi-like homologs (the first and last deciles of the distribution were removed before correlating; therefore, the significant correlation was not driven by those species). Especially *Folsomia candida*, *M. lignano*, *Cloeon dipterum*, and bdelloid rotifers (*A. ricciae*, *Rotaria socialis*, and *Didymodactylos carnosus*) were characterized by a strong expansion of both Piwi-like homologs and T3 genes (Figs. 3 and 5). For some of these species (*M. lignano* and Bdelloidea), expansions can be partially explained with WGD dynamics (see the first section of Discussion). However, most of the T3 gene radiations are broader than those of T0 and T1 within the same species and invoke further interpretations.

The piRNA pathway is extremely important in the genomic homeostasis of both germ and totipotent stem cells (such as planarian neoblasts or poriferan archaeocytes), preventing retrotransposable TE burst in dividing cells devoted to genetic transmission across generations (Juliano et al. 2010; Siomi et al. 2011). It is therefore unavoidable to interpret expansions and reduction of genes involved in this process in the light of lineage-specific TE composition and activity, as well as considering reproductive features.

In *M. lignano*, T3 genes are strikingly numerous, being the most numerous T3 gene set of all our dataset (63 genes; Fig. 3). Even considering the retention of the whole duplicated set, therefore, merely dividing by two to simulate pre-WGD condition, the number is still high and would fall in the first decile of the distribution. The massive expansion in the number of Piwi-like homologs (14 sequences) and T3 genes (19 belonging to OG164, 4 to TDRD12, 3 to TDRD9, and 36 belonging to nonannotated OGs) in *M. lignano* might be due to selection for TE regulation in a species were neoblasts represent the only dividing cells, being crucial for regeneration, tissue homeostasis, and asexual reproduction. Long terminal repeats (LTRs) retrotransposons, one of the TE classes whose mobility is directly controlled by piRNAs, represent the 21% of *M. lignano* genome (Wudarski et al. 2017), and in the present study, we could observe a high peak of very recent LTR retrotranspon activity (measured as frequency of lowly divergent elements; supplementary fig. S9, Supplementary Material online). The recent WGD might have induced an uncontrolled TE motility (Lien et al. 2016), and this might have promoted the selection for overnumerary copy numbers of both Piwi-like and T3 genes. Coherently, in *M. lignano,* most Piwi-like copies have extremely short branch lengths and can be divided into four clusters, each comprehending numerous paralogs very similar to each other (Fig. 5). Therefore, these Piwi-like genes are probably very recent and might be the sign of a presently occurring arms race driven by the highly dynamic *M. lignano* genomic environment.

Similar Piwi-like/T3 gene number expansions are present in Bdelloidea and the springtail *F. candida* (the latter having the outstanding number of 19 Piwi-like homologs and 45 T3 genes). Also, in these cases, the maintenance of overnumerary Piwi-like/T3 genes could have been selected for the regulation of TE activity. Indeed, these four species are parthenogenetic (bdelloids are apomictic; i.e. they lack meiosis), and it might be tempting to associate the T3 gene number expansion to the selection for an efficient TE control pathway in species that lack or have weaker sex-related defenses to mobile elements (e.g. genetic exchange among individuals and meiotic recombination). The consequences of asexuality and unisexuality on TE loads have always been of interest. It has been predicted that asexual populations that lacked efficient ways for controlling TE expansions should either accumulate them up to lineage extinction or lack TEs in the first place (Dolgin and Charlesworth 2006). However, this prediction was not always confirmed, and asexual or unisexual lineages usually show TE loads similar to closely related sexual taxa (see for instance Kraaijeveld et al. 2012; Bast et al. 2016; but see Jaron et al. 2021). In long-term asexual Bdelloidea, for example, relatively abundant, diversified, and recently active transposons and retrotransposons have been found (Nowell et al. 2021; but see also the recent LTR retrotransposon expansion observed in *Adineta vaga*: Kim et al. 2018). The same authors did not find any significant difference in respect to other Rotifera in terms of TE load, but they, however, found bdelloid-specific expansions of TE silencing pathways (Nowell et al. 2021). In our bdelloid species, and also in the other apomictic parthenogenetic species, the springtail *F. candida*, we could confirm an increase in Piwi-like/T3 genes, putatively selected to avoid detrimental effects of TE mobility in genomic systems that lack other effective molecular mechanisms of TE mobility prevention. In particular, the overnumerary copies of Piwi-like homologs in Bdelloidea and *F. candida* represent two clade-specific expansions (Fig. 5). However, the branches separating the paralogs are long and suggest a deep and old diversification, coherently with the ancient persistence of apomictic parthenogenesis in these species (tens of million years for bdelloid rotifers; Welch et al. 2009).

It is interesting to notice that another species with a conspicuously high number Piwi-like/T3 genes (12 Piwi-like; 39 T3) is the mayfly *C. dipterum.* In Ephemeroptera, parthenogenesis is supposed to be independently evolved in 12 different families, with different declensions (obligate, facultative, and spontaneous) and various percentages of occurrence, suggesting high evolvability of the trait in the clade (Liegeois et al. 2021). The estimate of occurrence in species studied for their reproductive mode is 47.8%, reaching ∼80% in some families like Baetidae, that includes *C. dipterum* (Liegeois et al. 2021). Even if the family of *C. dipterum* shows a strong propensity for parthenogenesis evolution (in both automictic and apomictic forms), the species has never been observed to reproduce parthenogenetically. It is tempting to interpret the here observed *C. dipterum* Piwi-like/T3 gene number expansion as a form of exaptation for parthenogenesis in such animals, being a potential advantage for TE load regulation.

### The Model of Stepwise Accumulation of N-t Secondary Structure Remains a Valid Hypothesis for Tudor Domain Evolution

The stepwise acquisition model of N-t extensions (Jin et al. 2009) theorizes that the ancestral Tudor domain would have been a T0 type, and our phylogenetic analyses based on Tudor domain sequences are consistent with this hypothesis. Indeed, beside the early divergence of PHF20-20L1, the T0 domain secondary structure is paraphyletic, with both T1, T2, and T3 domains nested within T0 clades. This pattern was consistent both in the NJ tree of the profiles and in all seven ML replicate trees (Fig. 4a and b; supplementary figs. S1 to S6, Supplementary Material online), and statistical support was provided by the ASR analyses that inferred T0 as the ancestral state with ∼1 probability for all ML replicates (supplementary table S4, Supplementary Material online).

According to the NJ tree of the profiles and most ML trees (four out of seven), the second Tudor domain of PHF20-20L1 is in a sister relationship with all other Tudor domains and has the lower distance from the remote PWWP homologs. Interestingly, previous works have found that the putatively Tudor C-terminal domain of ProQ in *Escherichia coli* has the highest structural similarity with the second Tudor of PHF20, with respect to all other PDB database hits (Gonzalez et al. 2017). Such bacterial ProQ Tudor-like domain was found so far exclusively in a limited set of γ-proteobacteria RNA chaperones, and previous authors speculatively suggested that its presence in prokaryotes might be the result of HGT rather than far vertical homology (Gonzalez et al. 2017; Liao and Smirnov 2023). However, structural similarities are the highest with the second Tudor domain of PHF20, and the fact that this domain is the closest one to the root and in a sister relationship with all other Tudor (in NJ tree and most ML ones) might be a hint for vertical homology from the bacterial Tudor-like domain. It might be interesting to more deeply characterize such similarities and discriminate between far homology and convergent evolution.

The prediction of the stepwise model of the emergence of the T2 domain from a single insertion event of a T1 domain within the fifth SN domain of SND1 followed by its co-option in the T3 domains (Jin et al. 2009) could not be strongly confirmed by our phylogenetic inference. Indeed, neither the NJ tree nor ML replicates consistently retrieved the expectation of a sister relationship of T1 domains with a T2 + T3 domain clade. The polyphyly of the N-t α-helix extensions could be explained either by a single acquisition of the N-t α-helix that might have been subsequently lost in the intercalated T0 domain clades or by the independent evolution of the α-helix in the progenitors of T1 and T2 domains. In the NJ tree, the putative independent losses or acquisitions would be two in both cases, but in the ML replicates, the situation is more convoluted and would require multiple events for both scenarios.

Another prediction of the stepwise model would be the sister relationship of T2 domains (those of SND1 proteins) and all T3 domains, but this was never retrieved in any of the ML replicates or the NJ tree: T2 domains always cluster within T3 domains in both methods. However, internal basal nodes of the T2 + T3 domains are very lowly supported and separated by very short branches (Fig. 4b) that probably reflect a relatively fast diversification. Collapsing nodes for UFB support still shows the monophyly of Tudor domains with N-t extension and highlights the difficulties in confidently solving their relationship. The evolutionarily fast T3 gene family expansion in Metazoa might have obscured the resolution power of phylogenetic inference, confounding the deep nodes of T2 + T3 domains and the inner relationships of the T3 domain clade but leaving as hints the much shorter and less supported internal branches than the rest of the tree.

Moreover, most T3 Tudor proteins were grouped within the large OG164 homology cluster, despite belonging to different orthologous genes, and T3 domains display an enormous range of different occurrences, from single copies to up to 19 multiple copies within the same protein (some cnidarian sequences included in OG164). These genes in OG164 almost perfectly coincided with the Tudor genes that were considered as metazoan innovations involved in the GMP program by Fierro-Constaín et al. (2017) (excluding TDRD9 and STK31 that constituted independent OGs). Curiously, the relationships among them could not be confidently solved and they were ascribed in the same homology cluster, suggesting a convoluted pattern of evolution that involved multiple duplications and likely a bricolage-like evolution by domain exon shuffling of the proteins that impeded the algorithm to distinguish orthologs. Their evolution might have been fast enough to impede distinguishing their reciprocal evolutionary relationships after hundreds of million years of sequence evolution (also considering the short length of the Tudor domain that led to infer phylogenetic relationships).

We believe that the previously proposed model elegantly based on structural similarities (the entangled association of the Tudor and the fifth SN domain of SND1; Jin et al. 2009) remains the strongest hypothesis. Some of the predictions of the model of Tudor evolution proposed by Jin et al. (2009) were here confirmed, while others could not be confidently supported due to lack of phylogenetic resolution. Nevertheless, they could not be excluded either, leaving the stepwise model as a valid hypothesis for the evolutionary pathways of the Tudor domain.

### Some Previously Considered Metazoa-Specific piRNA-Related Elements Were Present Also in Early-Branching Unicellular Holozoa

*Piwi* and other GMP genes (like *vasa* and *nanos*) were thought to be metazoan innovations evolved alongside multicellularity (Alié et al. 2015; Fierro-Constaín et al. 2017). Some Tudor genes, namely those belonging to the T3 structural group, are comprised among such innovations (Fierro-Constaín et al. 2017) as confirmed by the animal-wide distribution of these genes, comprising also the basal clades of Porifera, Cnidaria, and Ctenophora (Fig. 2).

Nonmetazoan eukaryotes were thought to completely lack T3 genes, but some were recently annotated in choanoflagellates of the *Salpingoeca* order (Bell et al. 2020). With the present study, we expand the number of unicellular Holozoa that present T3 domains, annotating them in two Ichthyosporea species, that represent early branching holozoan clades. The lack of homology between Bell et al. (2020) T3 + DCD proteins and any sequence of our holozoan proteomes, and the differences in domain composition between the two ichthyosporean T3 genes, complicate the scenario. Indeed, considering the ML analyses and the different domain composition of *I. hoferi* and *C. perkinsii* T3 genes, a plausible hypothesis is that they represent independent co-options of T3 domains. *Chromosphaera perkinsii* T3 domains cluster scattered among metazoan ones, and the belonging protein clustered in a separated OG with some Tardigrada sequences, possibly meaning that it is the result of HGT. *Ichthyophonus hoferi* T3 domain, on the other hand, belonged to a protein included by OrthoFinder in the “noisy” OG164, and in the ML tree, it clustered in a polytomy with T2 domains belonging to SND1 proteins (Fig. 4b). *Ichthyophonus hoferi* T3 domain indeed represents a good candidate for the early evolution of the piRNA silencing toolkit, even more than the choanoflagellate sequences annotated by Bell et al. (2020). Indeed, while choanoflagellates appear to completely lack Piwi-like homologs, we found that *I. hoferi* has one whose position in a sister relationship with all animal Piwi-like proteins could not be refused (based on Piwi + PAZ domains). Moreover, *I. hoferi* Piwi-like sequence presents multiple RG motifs in its N-t position, comprising many positions conserved in most animal sequences (supplementary fig. S7, Supplementary Material online). These Piwi RG motifs are those that get methylated and then recognized by the sDMA-binding activity of T3 domains (Liu et al. 2010), meaning that these might be potential binding sites for *I. hoferi* T3 protein.

Our data predate the emergence of both T3 gene elements and Piwi-like ones to early diverging Holozoa, suggesting that at least part of a potentially functional piRNA genetic toolkit was shared by the common ancestor of all Holozoa and is still present in some ichthyosporean species. These genes might have been subsequently lost in most unicellular phyla while they expanded in Metazoa, in which they were co-opted for functions strictly related to multicellularity (i.e. germline establishment). However, by proposing *I. hoferi*, *C. perkinsii*, and Choanoflagellata T3 genes as independent evolutions, we delineate a scenario where the co-option of Tudor domains is a relatively frequent event, and the metazoan emergence might simply represent an additional independent co-option. The future inclusion of additional genomic resources and works focused on unicellular holozoan might provide a better understanding of the convoluted evolutionary dynamics of T3 domains.

## Conclusions

In the present study, we conducted a wide-scale evolutionary survey of the Tudor domain-containing proteins in Metazoa and their closely related unicellular holozoan relatives. We aimed to characterize the distribution of the gene family in the different animal phyla, trying to explain the evolutionary forces that shaped such variability and to test a previously proposed model of the evolution of the Tudor domain secondary structure.

Our observations suggest that some major and potentially functional elements of the piRNA pathway were already present in the genomic landscape of the first Holozoa. Their massive and relatively quick expansion at the root of animals provided the genetic potential for their co-option in finely-tuned multicellularity-related pathways.

We could observe an outstanding variability of Tudor gene numbers across phyla and species. Overall, Tudor gene number extreme variations were associated to whole-genome expansion and reduction, probably due to their epigenetic regulatory roles. However, T3 gene set dynamics were largely explained by specific evolutionary patterns of the piRNA pathway, with severe reduction or complete loss in species that lacked Piwi-like homologs, and conspicuous expansions in species in which selection for stringent TE regulatory dynamics was/is advantageous (e.g. parthenogenetic species).

With phylogenetic tools based on Tudor domain sequences, we tested the so-called stepwise model of Tudor N-t structure acquisition, confirming the T0 domain type (no N-t extension) as the ancestral state. The further predictions of the model, i.e. a single acquisition of the N-t α-helix and the co-option of the T2 domain of SND1 in the T3 gene metazoan radiation, were not rejected by our analysis, but their support was nevertheless slight.

## Materials and Methods

### Dataset

We downloaded from online-available annotated genomes 112 species proteomes, covering 21 metazoan phyla and four major taxonomic groups of unicellular Holozoa (supplementary table S1, Supplementary Material online). The choice of proteomes was driven to maximize phylogenetic representativeness, including all available proteomes for lowly covered phyla, and no more than ten species for highly represented taxonomic groups like vertebrates and insects. We collapsed the proteomes keeping exclusively the longest isoform for each gene (when unique gene identifiers were present: roughly two-thirds of the dataset).

### Tudor Protein Identification and Tudor Domain Extraction

To identify the Tudor proteins in the proteomes, we first inferred homology clusters between all proteomes with OrthoFinder v.2.3.11 (with *--ultra-sensitive* parameter). We then annotated all OrthoFinder's OGs that included at least one Tudor domain, cross-checking with the domain annotation performed on the whole dataset with InterProScan (domain annotation *e*-value cutoff: 10^−5^; the InterProScan codes that we used are in supplementary table S7, Supplementary Material online).

For those OGs that collected at least ten sequences belonging to at least 2 species and that contained at least 10% of Tudor proteins, we implemented the raw InterProScan annotation with iterative within-OG HMMER profile searches. For each OG, we built a profile on the alignments of their Tudor domains (sequences aligned with MAFFT-DASH v7.471, *--global-pair* algorithm; Katoh and Standley 2013; profiles built with *hmmbuild* by HMMER v3.2.1), and we run it back on all sequences of its belonging OG (*hmmsearch* by HMMER). If additional domains were retrieved, they were included in the construction of a new profile for a subsequent iteration, and so on until no further sequences were found. We performed these iterations with different *hmmbuild -symfrac* parameter values (namely 30%, 50%, and 70%; threshold for the percentage of gaps in a position; supplementary table S8, Supplementary Material online): we then kept results for the value that needed the lowest number of iterations to reach plateau to minimize the risk of flanking position inclusions. When the annotation pipeline did not automatically separate multiple Tudor domains occurring in the same sequences (for some cases where consecutive domains were very close), we did it manually using the positional information on *H. sapiens* (namely for SGF29, Espinola-Lopez and Tan 2021; TP53BP1, Charier et al. 2004; UHFR1, Kori et al. 2019).

The secondary structures of all Tudor domains of our dataset were predicted with SSpro v6.0 as implemented in SCRATCH-1D v2.0 (Cheng et al. 2005). We then manually annotated the presence of α-helices and β-strands in the proximity of the Tudor domain core (five β-strands), and each Tudor gene was assigned to a different class based on their domain structure: T0 for no N-t extension; T1 for N-t α-helix; and T2 and T3 for N-t α-helix and two β-strands (T2 group referring to SND1 Tudor domain only due to functional reasons and previous classifications; Jin et al. 2009).

### Statistical Analyses

Statistical analyses were performed correlating the number of Tudor genes for each species with other genetic and genomic variables. We considered for this analysis all Tudor genes, therefore, including both those that underwent the curated iterative annotation for a subset of OGs and all other Tudor genes identified by the raw InterProScan domain annotation.

First, we checked for any possible bias due to the quality of genome annotation, but we found no correlations between the BUSCO completeness (Metazoa database, v5; Manni et al. 2021) and the total number of genes. We then performed correlations considering separately the subsets corresponding to the three Tudor domain structural types (T2 not considered). The variables investigated were as follows: the number of Piwi*-*like homologs (sequences with Piwi and PAZ domains in the OG that included Piwi, Ago3, and Aub of *D. melanogaster* and PiwiL1-2-3-4 of *H. sapiens*); the number of Ago-like homologs (OG containing Ago1-2 of *D. melanogaster* and Ago1-2-4 of *H. sapiens*); the number of all protein-coding genes; the genome size (in terms of *C*-value; retrieved from NCBI's genomesize.com or from the specific databases from which the proteome was collected); the gene density (number of protein-coding genes over genome size); and the splicing index (number of all protein products over the number of protein-coding genes, exclusively for those genomes for which isoform annotation was present: roughly two-thirds). Given that the distribution of Tudor genes in our species was highly variable, with cases of extreme reduction and expansion, to perform correlation tests, we removed the edges of the distribution (below the 10th and above the 90th percentiles of the total number of Tudor genes: below 11 and over 45) to avoid the statistics being driven by them.

We corrected for phylogenetic biases by calculating phylogenetic independent contrasts (PICs; calculated with *pic* function of *ape* package in R; Felsenstein 1985). The phylogenetic tree we used to calculate PICs was built on 333 BUSCO v5 genes (Metazoa database) present in more than 90% of the species (alignment: MAFFT; trimming: BMGE; model selection: IQTREE's ModelFinder; branch length calculation: RAxML-NG, Kozlov et al. 2019; topology was fixed and based on the literature: see Extended_reference.pdf in Supplementary material for references; alignment and tree provided in Supplementary material). For each pair of tested PICs, we fitted a linear model with *lm* function in R (“*y* ∼ *x* − 1” to force it through the origin given that the sign of the contrasts calculated with *pic* functions is arbitrary). *P*-values were adjusted with the “fdr” method of *p.adjust* function of R. Since we were interested in the metazoan variability of the Tudor family, and to avoid biases due to the smaller genome sizes and lower gene content of nonmetazoan Holozoa, we performed all correlations excluding unicellular Holozoa. All noncorrected numerical values are present in supplementary table S2, Supplementary Material online.

### Tudor Domain Phylogenetic Inference

To explore the evolutionary history of the Tudor domain, we considered only domain sequences from Tudor OGs for which the iterative HMMER annotation was performed. Moreover, to reduce complexity and avoid misannotations, we excluded sequences with length lower than 45 amino acids (roughly 3/4 of the T0 Tudor domain length). For the noisy OG164, we used DISCO to subsample the OG in orthology groups. We aligned the whole-length proteins with MAFFT and ran an IQTREE tree inference. We then fed the OG164 tree to DISCO that divides it into subtrees coherent with a reference species tree (built based on the literature). We obtained eight subclades of orthology that were treated separately in the phylogenetic analyses.

Tudor domains of each OG were aligned with MAFFT-DASH (--*globalpair*), using tertiary structures from online databases (Rozewicki et al. 2019). We evaluated the alignment with the Transitive Consistency Score (TCS) calculated by TCOFFEE (Chang et al. 2014) and excluded sequences with a score lower than 50 (this step was performed iteratively until all sequences had a TCS higher or equal to 50).

As a first phylogenetic approach, we used HMM profiles to infer the evolutionary relationships between the Tudor domains, simplifying the dataset while attempting to preserve as much information as possible about sequence variability. We built an HMM profile for each Tudor domain belonging to each OG (*hmmbuild* by HMMER), and a NJ profile tree was inferred with pHMM-Tree, based on a distance matrix calculated with the PRC algorithm. When a gene contained multiple Tudor domains, we considered each separately (e.g. for the first and second Tudor of KDM4A/B/C, two different profiles were built). When domains could not be confidently split due to uncertain homology relationships (e.g. in OG0009555, some species contained two domains and some one), a single profile for all Tudor domains was built. The HMMER profile of the PWWP domain was used as outgroup (domains extracted from the more numerous OG whose sequences included PWWP domains, following the same procedure of Tudor domain profiles).

We also inferred ML replicate trees of the domain sequences with IQ-TREE. Given the high number of sequences and the low length of the domain, we subsampled the dataset in order to reduce the enormous tree space that the software would have explored. For each Tudor domain, we kept only the 50% of sequences with higher similarity to the OG's HMMER profile (*hmmsearch* of profile against sequences). Moreover, we excluded from the ML inference the noisy OG164. Considering the complex evolution that probably lies behind these multidomain proteins and considering that they consisted in almost half of the total number of Tudor domains (therefore consistently expanding the tree space), we decided to remove them. The final sequences (1,435 Tudor domains) were aligned with MAFFT-DASH (--*globalpair* algorithm) and trimmed with BMGE v1.12 (*-b 1 -g 0.999 -h 0.6*; Criscuolo and Gribaldo 2010), and ten IQ-TREE replicates were run (with 1000 UFB replicates each), forcing PWWP monophyly. Model testing was performed by ModelFinderProtein as implemented by IQ-TREE: we tested LG, WAG, and JTT including all combinations of models of rate heterogeneity across sites and with or without empirical amino acid frequencies; mixture models C10-60, LG4X, and LG4M were included in the model testing. The best-fitting model according to Bayesian information criterion was LG + R7, which was used for all ten replicates. The likelihoods of the ten replicate ML trees were evaluated with the topology tests as implemented by IQ-TREE (approximately unbiased test: Shimodaira 2002; bootstrap proportion using RELL method; Kishino–Hasegawa test: 1989; Kishino et al. 1990; Shimodaira–Hasegawa test: 1999; expected likelihood weight: Strimmer and Rambaut 2002).

Different models of character (T0/T1/T2 + T3) evolution were tested for all ten ML replicates with the *fitDiscrete* function of R geiger package (ER, SYM, and ARD models were tested). ASR analyses were conducted with the selected model (lowest corrected Akaike information criterion) with the *corHMM* function of R corHMM package.

### Ichthyosporean T3 and Piwi-Like Protein Domains Phylogenetic Inference

Due to our filters (see previous subsection), the ML trees inferred on the Tudor domain alignment did not include neither *I. hoferi* T3 gene (belonging to the excluded OG164) nor the *C. perkinsii* one (belonging to a small OG not included in the analysis). To get insight on their evolutionary history, we extracted their belonging T3 domains and realigned them with all other Tudor (MAFFT-DASH *--add* option). We then built ten ML trees with IQTREE, forcing the best previously obtained topology and allowing for the positions of ichthyosporean sequences to be freely placed in the tree. To test a priori topologies, we built trees with sequences of SND1 and the eight subclades of OG164 (MAFFT-DASH + BMGE + IQTREE): ten replicates with free topology (only imposing monophyly of SND1) and ten replicates with *I. hoferi* T3 domain in a sister relationship with all other OG164 domains. Tree topologies were then evaluated with all topology tests implemented by IQTREE.

For Piwi-like homologs, we implemented the Piwi domain annotation within the OG using the same iterative HMMER-profile pipeline used for the Tudor domain extraction. To build ML phylogenetic trees, we used a concatenated alignment of Piwi and PAZ domain amino acid sequences aligned with MAFFT-DASH (*--global-pair*). Model testing was performed with ModelFinderProtein as implemented in IQTREE on the partitions corresponding to the two domains, including all models used for the Tudor phylogeny: LG + C50 + F and LG + C60 + F were retrieved as best models for Piwi and PAZ partitions, respectively. We ran ten replicates, and trees were rooted with 24 sequences belonging to Ago-like homologs (one for each phylum). To further test the relationship of the two ichthyosporean sequences, we ran ten replicates forcing them to be in a sister relationship with all Metazoa, ten replicates forcing only *C. perkinsii* as sister, and ten replicates forcing only *I. hoferi*. Tree topologies were then evaluated with all topology tests implemented by IQTREE.

## Supplementary Material

evaf051_Supplementary_Data

## Data Availability

Output result files (each OG MAFFT-DASH alignment and HMM profile + all ten ML replicate topologies + ichthyosporean T3 sequences + Piwi-like sequences + OG164 analyses output files + tree inference for PIC calculation) are available at https://github.com/GiovanniPiccinini/Tudor_paper.git. For any other request, please contact the corresponding author.

## References

[evaf051-B1] Alié A, Hayashi T, Sugimura I, Manuel M, Sugano W, Mano A, Satoh N, Agata K, Funayama N. The ancestral gene repertoire of animal stem cells. Proc Natl Acad Sci U S A. 2015:112(51):E7093–E7100. 10.1073/pnas.1514789112. 26644562 PMC4697369

[evaf051-B2] Bast J, Schaefer I, Schwander T, Maraun M, Scheu S, Kraaijeveld K. No accumulation of transposable elements in asexual arthropods. Mol Biol Evol. 2016:33(3):697–706. 10.1093/molbev/msv261. 26560353 PMC4760076

[evaf051-B3] Bell RT, Wolf YI, Koonin EV. Modified base-binding EVE and DCD domains: striking diversity of genomic contexts in prokaryotes and predicted involvement in a variety of cellular processes. BMC Biol. 2020:18(1):159. 10.1186/s12915-020-00885-2. 33148243 PMC7641849

[evaf051-B4] Blaxter M, Koutsovoulos G. The evolution of parasitism in Nematoda. Parasitology. 2015:142(Suppl 1):S26–S39. 10.1017/S0031182014000791. 24963797 PMC4413787

[evaf051-B5] Bleidorn C . Recent progress in reconstructing lophotrochozoan (spiralian) phylogeny. Org Divers Evol. 2019:19(4):557–566. 10.1007/s13127-019-00412-4.

[evaf051-B6] Botuyan MV, Mer G. Tudor domains as methyl-lysine and methyl-arginine readers. In: Chromatin signaling and diseases. Elsevier Inc.; 2016. p. 149–165.

[evaf051-B7] Caudy AA, Ketting RF, Hammond SM, Denli AM, Bathoorn AM, Tops BB, Silva JM, Myers MM, Hannon GJ, Plasterk RH. A micrococcal nuclease homologue in RNAi effector complexes. Nature. 2003:425(6956):411–414. 10.1038/nature01956. 14508492

[evaf051-B8] Chang JM, Di Tommaso P, Notredame C. TCS: a new multiple sequence alignment reliability measure to estimate alignment accuracy and improve phylogenetic tree reconstruction. Mol Biol Evol. 2014:31(6):1625–1637. 10.1093/molbev/msu117. 24694831

[evaf051-B9] Charier G, Couprie J, Alpha-Bazin B, Meyer V, Quéméneur E, Guérois R, Callebaut I, Gilquin B, Zinn-Justin S. The Tudor tandem of 53BP1: a new structural motif involved in DNA and RG-rich peptide binding. Structure. 2004:12(9):1551–1562. 10.1016/j.str.2004.06.014. 15341721

[evaf051-B10] Chen C, Nott TJ, Jin J, Pawson T. Deciphering arginine methylation: Tudor tells the tale. Nat Rev Mol Cell Biol. 2011:12(10):629–642. 10.1038/nrm3185. 21915143

[evaf051-B11] Chen F, Luo M, Lai F, Yu C, Cheng H, Zhou R. Biased duplications and loss of members in Tdrd family in teleost fish. J Exp Zool B Mol Dev Evol. 2017:328(8):727–736. 10.1002/jez.b.22757. 28660752

[evaf051-B12] Cheng J, Sweredoski MJ, Baldi P. Accurate prediction of protein disordered regions by mining protein structure data. Data Min Knowl Discov. 2005:11(3):213–222. 10.1007/s10618-005-0001-y.

[evaf051-B13] Cheng P, Huang Y, Du H, Li C, Lv Y, Ruan R, Ye H, Bian C, You X, Xu J, et al Draft genome and complete *Hox*-cluster characterization of the sterlet (*Acipenser ruthenus*). Front Genet. 2019:10:776. 10.3389/fgene.2019.00776. 31543900 PMC6739705

[evaf051-B14] Côté J, Richard S. Tudor domains bind symmetrical dimethylated arginines. J Biol Chem. 2005:280(31):28476–28483. 10.1074/jbc.M414328200. 15955813

[evaf051-B15] Criscuolo A, Gribaldo S. BMGE (block mapping and gathering with entropy): a new software for selection of phylogenetic informative regions from multiple sequence alignments. BMC Evol Biol. 2010:10(1):210. 10.1186/1471-2148-10-210. 20626897 PMC3017758

[evaf051-B16] Dolgin ES, Charlesworth B. The fate of transposable elements in asexual populations. Genetics. 2006:174(2):817–827. 10.1534/genetics.106.060434. 16888330 PMC1602064

[evaf051-B17] Du K, Stöck M, Kneitz S, Klopp C, Woltering JM, Adolfi MC, Feron R, Prokopov D, Makunin A, Kichigin I, et al The sterlet sturgeon genome sequence and the mechanisms of segmental rediploidization. Nat Ecol Evol. 2020:4(6):841–852. 10.1038/s41559-020-1166-x. 32231327 PMC7269910

[evaf051-B18] Eddy SR . Accelerated profile HMM searches. PLoS Comput Biol. 2011:7(10):e1002195. 10.1371/journal.pcbi.1002195. 22039361 PMC3197634

[evaf051-B19] Emms DM, Kelly S. OrthoFinder: phylogenetic orthology inference for comparative genomics. Genome Biol. 2019:20(1):238. 10.1186/s13059-019-1832-y. 31727128 PMC6857279

[evaf051-B20] Espinola-Lopez JM, Tan S. The Ada2/Ada3/Gcn5/Sgf29 histone acetyltransferase module. Biochim Biophys Acta Gene Regul Mech. 2021:1864(2):194629. 10.1016/j.bbagrm.2020.194629. 32890768 PMC8351874

[evaf051-B21] Felsenstein J . Phylogenies and the comparative method. Am Nat. 1985:125(1):1–15. 10.1086/284325. 31094602

[evaf051-B22] Fierro-Constaín L, Schenkelaars Q, Gazave E, Haguenauer A, Rocher C, Ereskovsky A, Borchiellini C, Renard E. The conservation of the germline multipotency program, from sponges to vertebrates: a stepping stone to understanding the somatic and germline origins. Genome Biol Evol. 2017:9(3):474–488. 10.1093/gbe/evw289. 28082608 PMC5381599

[evaf051-B23] Fontenla S, Rinaldi G, Tort JF. Lost and found: Piwi and Argonaute pathways in flatworms. Front Cell Infect Microbiol. 2021:11:653695. 10.3389/fcimb.2021.653695. 34123869 PMC8191739

[evaf051-B24] Freeling M, Thomas BC. Gene-balanced duplications, like tetraploidy, provide predictable drive to increase morphological complexity. Genome Res. 2006:16(7):805–814. 10.1101/gr.3681406. 16818725

[evaf051-B25] Gao X, Zhao X, Zhu Y, He J, Shao J, Su C, Zhang Y, Zhang W, Saarikettu J, Silvennoinen O, et al Tudor staphylococcal nuclease (Tudor-SN) participates in small ribonucleoprotein (snRNP) assembly via interacting with symmetrically dimethylated Sm proteins. J Biol Chem. 2012:287(22):18130–18141. 10.1074/jbc.M111.311852. 22493508 PMC3365748

[evaf051-B26] Gao XD, Tachikawa H, Sato T, Jigami Y, Dean N. Alg14 recruits AlG13 to the cytoplasmic face of the endoplasmic reticulum to form a novel bipartite UDP-N-acetylglucosamine transferase required for the second step of N-linked glycosylation. J Biol Chem. 2005:280(43):36254–36262. 10.1074/jbc.M507569200. 16100110

[evaf051-B27] Glenn LE, Searles LL. Distinct domains mediate the early and late functions of the *Drosophila* ovarian tumor proteins. Mech Dev. 2001:102(1-2):181–191. 10.1016/S0925-4773(01)00314-8. 11287191

[evaf051-B28] Gonzalez GM, Hardwick SW, Maslen SL, Skehel JM, Holmqvist E, Vogel J, Bateman A, Luisi BF, Broadhurst RW. Structure of the *Escherichia coli* ProQ RNA-binding protein. RNA. 2017:23(5):696–711. 10.1261/rna.060343.116. 28193673 PMC5393179

[evaf051-B29] Grau-Bové G, Torruella G, Donachie S, Suga H, Leonard G, Richards TA, Ruiz-Trillo I. Dynamics of genomic innovation in the unicellular ancestry of animals. eLife. 2017:6:e26036. 10.7554/eLife.26036. 28726632 PMC5560861

[evaf051-B30] Gutierrez-Beltran E, Denisenko TV, Zhivotovsky B, Bozhkov PV. Tudor staphylococcal nuclease: biochemistry and functions. Cell Death Differ. 2016:23(11):1739–1748. 10.1038/cdd.2016.93. 27612014 PMC5071578

[evaf051-B31] Huo L, Zhang H, Huo X, Yang Y, Li X, Yin Y. pHMM-Tree: phylogeny of profile hidden Markov models. Bioinformatics. 2017:33(7):1093–1095. 10.1093/bioinformatics/btw779. 28062446 PMC5860389

[evaf051-B32] Hur JH, van Doninck K, Mandigo ML, Meselson M. Degenerate tetraploidy was established before bdelloid rotifer families diverged. Mol Biol Evol. 2009:26(2):375–383. 10.1093/molbev/msn260. 18996928

[evaf051-B33] Jaron KS, Bast J, Nowell RW, Ranallo-Benavidez TR, Robinson-Rechavi M, Schwander T. Genomic features of parthenogenetic animals. J Hered. 2021:112(1):19–33. 10.1093/jhered/esaa031. 32985658 PMC7953838

[evaf051-B34] Jiang WK, Liu YL, Xia EH, Gao LZ. Prevalent role of gene features in determining evolutionary fates of whole-genome duplication duplicated genes in flowering plants. Plant Physiol. 2013:161(4):1844–1861. 10.1104/pp.112.200147. 23396833 PMC3613460

[evaf051-B35] Jin J, Xie X, Chen C, Park JG, Stark C, James DA, Olhovsky M, Linding R, Mao Y, Pawson T. Eukaryotic protein domains as functional units of cellular evolution. Sci Signal. 2009:2(98):ra76. 10.1126/scisignal.2000546. 19934434

[evaf051-B36] Jones P, Binns D, Chang HY, Fraser M, Li W, McAnulla C, McWilliam H, Maslen J, Mitchell A, Nuka G, et al InterProScan 5: genome-scale protein function classification. Bioinformatics. 2014:30(9):1236–1240. 10.1093/bioinformatics/btu031. 24451626 PMC3998142

[evaf051-B37] Juliano C, Wang J, Lin H. Uniting germline and stem cells: the function of Piwi proteins and the piRNA pathway in diverse organisms. Annu Rev Genet. 2011:45(1):447–469. 10.1146/annurev-genet-110410-132541. 21942366 PMC3832951

[evaf051-B38] Juliano CE, Swartz SZ, Wessel GM. A conserved germline multipotency program. Development. 2010:137(24):4113–4126. 10.1242/dev.047969. 21098563 PMC2990204

[evaf051-B39] Katoh K, Standley DM. MAFFT multiple sequence alignment software version 7: improvements in performance and usability. Mol Biol Evol. 2013:30(4):772–780. 10.1093/molbev/mst010. 23329690 PMC3603318

[evaf051-B40] Kim HS, Lee BY, Han J, Jeong CB, Hwang DS, Lee MC, Kang HM, Kim DH, Kim HJ, Papakostas S, et al The genome of the freshwater monogonont rotifer *Brachionus calyciflorus*. Mol Ecol Resour. 2018:18(3):646–655. 10.1111/1755-0998.12768. 29451365

[evaf051-B41] Kishino H, Hasegawa M. Evaluation of the maximum likelihood estimate of the evolutionary tree topologies from DNA sequence data, and the branching order in Hominoidea. J Mol Evol. 1989:29(2):170–179. 10.1007/BF02100115. 2509717

[evaf051-B42] Kishino H, Miyata T, Hasegawa M. Maximum likelihood inference of protein phylogeny and the origin of chloroplasts. J Mol Evol. 1990:31(2):151–160. 10.1007/BF02109483.

[evaf051-B43] Kolb SJ, Battle DJ, Dreyfuss G. Molecular functions of the SMN complex. J Child Neurol. 2007:22(8):990–994. 10.1177/0883073807305666. 17761654

[evaf051-B44] Kori S, Ferry L, Matano S, Jimenji T, Kodera N, Tsusaka T, Matsumura R, Oda T, Sato M, Dohmae N, et al Structure of the UHRF1 tandem Tudor domain bound to a methylated non-histone protein, LIG1, reveals rules for binding and regulation. Structure. 2019:27(3):485–496.e7. 10.1016/j.str.2018.11.012. 30639225

[evaf051-B45] Kozlov AM, Darriba D, Flour T, Morel B, Stamatakis A. RAxML-NG: a fast, scalable and user-friendly tool for maximum likelihood phylogenetic inference. Bioinformatics. 2019:35(21):4453–4455. 10.1093/bioinformatics/btz305. 31070718 PMC6821337

[evaf051-B46] Kraaijeveld K, Zwanenburg B, Hubert B, Vieira C, de Pater S, van Alphen JJM, den Dunnen JT, de Knijff P. Transposon proliferation in an asexual parasitoid. Mol Ecol. 2012:21(16):3898–3906. 10.1111/j.1365-294X.2012.5582.x. 22548357

[evaf051-B47] Laumer CE, Fernández R, Lemer S, Combosch D, Kocot KM, Riesgo A, Andrade SCS, Sterrer W, Sørensen MV, Giribet G. Revisiting metazoan phylogeny with genomic sampling of all phyla. Proc Biol Sci. 2019:286(1906):20190831. 10.1098/rspb.2019.0831. 31288696 PMC6650721

[evaf051-B48] Liao Z, Smirnov A. Fino/ProQ-family proteins: an evolutionary perspective. Biosci Rep. 2023:43(3):BSR20220313. 10.1042/BSR20220313. 36787218 PMC9977716

[evaf051-B49] Liegeois M, Sartori M, Schwander T. Extremely widespread parthenogenesis and a trade-off between alternative forms of reproduction in mayflies (Ephemeroptera). J Hered. 2021:112(1):45–57. 10.1093/jhered/esaa027. 32918457 PMC7953839

[evaf051-B50] Lien S, Koop BF, Sandve SR, Miller JR, Kent MP, Nome T, Hvidsten TR, Leong JS, Minkley DR, Zimin A, et al The Atlantic salmon genome provides insights into rediploidization. Nature. 2016:533(7602):200–205. 10.1038/nature17164. 27088604 PMC8127823

[evaf051-B51] Lim RS, Kai T. A piece of the pi(e): the diverse roles of animal piRNAs and their PIWI partners. Semin Cell Dev Biol. 2015:47-48:17–31. 10.1016/j.semcdb.2015.10.025. 26582251

[evaf051-B52] Liu K, Chen C, Guo Y, Lam R, Bian C, Xu C, Zhao DY, Jin J, MacKenzie F, Pawson T, et al Structural basis for recognition of arginine methylated Piwi proteins by the extended Tudor domain. Proc Natl Acad Sci U S A. 2010:107(43):18398–18403. 10.1073/pnas.1013106107. 20937909 PMC2972943

[evaf051-B53] Liu Z, Liu S, Guo S, Lu W, Zhang Q, Cheng J. Evolutionary dynamics and conserved function of the Tudor domain-containing (TDRD) proteins in teleost fish. Mar Life Sci Technol. 2022:4(1):18–30. 10.1007/s42995-021-00118-7. 37073353 PMC10077171

[evaf051-B54] López-Escardó D, Grau-Bové X, Guillaumet-Adkins A, Gut M, Sieracki ME, Ruiz-Trillo I. Reconstruction of protein domain evolution using single-cell amplified genomes of uncultured choanoflagellates sheds light on the origin of animals. Philos Trans R Soc Lond B. 2019:374(1786):20190088. 10.1098/rstb.2019.0088. 31587642 PMC6792448

[evaf051-B55] Lu R, Wang GG. Tudor: a versatile family of histone methylation ‘readers’. Trends Biochem Sci. 2013:38(11):546–555. 10.1016/j.tibs.2013.08.002. 24035451 PMC3830939

[evaf051-B56] Manni M, Berkeley MR, Seppey M, Simão FA, Zdobnov EM. BUSCO update: novel and streamlined workflows along with broader and deeper phylogenetic coverage for scoring of eukaryotic, prokaryotic, and viral genomes. Mol Biol Evol. 2021:38(10):4647–4654. 10.1093/molbev/msab199. 34320186 PMC8476166

[evaf051-B57] Maurer-Stroh S, Dickens NJ, Hughes-Davies L, Kouzarides T, Eisenhaber F, Ponting CP. The Tudor domain ‘Royal Family’: Tudor, plant Agenet, Chromo, PWWP and MBT domains. Trends Biochem Sci. 2003:28(2):69–74. 10.1016/S0968-0004(03)00004-5. 12575993

[evaf051-B58] Minh BQ, Schmidt HA, Chernomor O, Schrempf D, Woodhams MD, von Haeseler A, Lanfear R. IQ-TREE 2: new models and efficient methods for phylogenetic inference in the genomic era. Mol Biol Evol. 2020:37(5):1530–1534. 10.1093/molbev/msaa015. 32011700 PMC7182206

[evaf051-B59] Nowell RW, Almeida P, Wilson CG, Smith TP, Fontaneto D, Crisp A, Micklem G, Tunnacliffe A, Boschetti C, Barraclough TG. Comparative genomics of bdelloid rotifers: insights from desiccating and nondesiccating species. PLoS Biol. 2018:16(4):e2004830. 10.1371/journal.pbio.2004830. 29689044 PMC5916493

[evaf051-B60] Nowell RW, Wilson CG, Almeida P, Schiffer PH, Fontaneto D, Becks L, Rodriguez F, Arkhipova IR, Barraclough TG. Evolutionary dynamics of transposable elements in bdelloid rotifers. eLife. 2021:10:e63194. 10.7554/eLife.63194. 33543711 PMC7943196

[evaf051-B61] Pauli D, Oliver B, Mahowald AP. The role of the ovarian tumor locus in *Drosophila melanogaster* germ line sex determination. Development. 1993:119(1):123–134. 10.1242/dev.119.1.123. 8275850

[evaf051-B62] Pek JW, Anand A, Kai T. Tudor domain proteins in development. Development. 2012:139(13):2255–2266. 10.1242/dev.073304. 22669818

[evaf051-B63] Rozewicki J, Li S, Amada KM, Standley DM, Katoh K. MAFFT-DASH: integrated protein sequence and structural alignment. Nucleic Acids Res. 2019:47(W1):W5–W10. 10.1093/nar/gkz342. 31062021 PMC6602451

[evaf051-B64] Selenko P, Sprangers R, Stier G, Bühler D, Fischer U, Sattler M. SMN Tudor domain structure and its interaction with the Sm proteins. Nat Struct Biol. 2001:8(1):27–31. 10.1038/83014. 11135666

[evaf051-B65] Sémon M, Wolfe KH. Consequences of genome duplication. Curr Opin Genet Dev. 2007:17(6):505–512. 10.1016/j.gde.2007.09.007. 18006297

[evaf051-B66] Shimodaira H . An approximately unbiased test of phylogenetic tree selection. Syst Biol. 2002:51(3):492–508. 10.1080/10635150290069913. 12079646

[evaf051-B67] Shimodaira H, Hasegawa M. Multiple comparisons of log-likelihoods with applications to phylogenetic inference. Mol Biol Evol. 1999:16(8):1114–1116. 10.1093/oxfordjournals.molbev.a026201.

[evaf051-B68] Siomi MC, Mannen T, Siomi H. How does the royal family of Tudor rule the PIWI-interacting RNA pathway? Genes Dev. 2010:24(7):636–646. 10.1101/gad.1899210. 20360382 PMC2849120

[evaf051-B69] Siomi MC, Sato K, Pezic D, Aravin AA. PIWI-interacting small RNAs: the vanguard of genome defence. Nat Rev Mol Cell Biol. 2011:12(4):246–258. 10.1038/nrm3089. 21427766

[evaf051-B70] Skinner DE, Rinaldi G, Koziol U, Brehm K, Brindley PJ. How might flukes and tapeworms maintain genome integrity without a canonical piRNA pathway? Trends Parasitol. 2014:30(3):123–129. 10.1016/j.pt.2014.01.001. 24485046 PMC3941195

[evaf051-B71] Strimmer K, Rambaut A. Inferring confidence sets of possibly misspecified gene trees. Proc Biol Sci. 2002:269(1487):137–142. 10.1098/rspb.2001.1862. 11798428 PMC1690879

[evaf051-B72] Tsai IJ, Zarowiecki M, Holroyd N, Garciarrubio A, Sánchez-Flores A, Brooks KL, Tracey A, Bobes RJ, Fragoso G, Sciutto E, et al The genomes of four tapeworm species reveal adaptations to parasitism. Nature. 2013:496(7443):57–63. 10.1038/nature12031. 23485966 PMC3964345

[evaf051-B73] Viney M . The genomic basis of nematode parasitism. Brief Funct Genomics. 2018:17(1):8–14. 10.1093/bfgp/elx010. 28472353 PMC5886223

[evaf051-B74] Weinstein SB, Kuris AM. Independent origins of parasitism in Animalia. Biol Lett. 2016:12(7):20160324. 10.1098/rsbl.2016.0324. 27436119 PMC4971171

[evaf051-B75] Welch DBM, Ricci C, Meselson M. Bdelloid rotifers: progress in understanding the success of an evolutionary scandal. In: Lost sex: the evolutionary biology of parthenogenesis. Springer; 2009. p. 259–279. 10.1007/978-90-481-2770-2_13.

[evaf051-B76] Willson J, Roddur MS, Liu B, Zaharias P, Warnow T. DISCO: species tree inference using multicopy gene family tree decomposition. Syst Biol. 2022:71(3):610–629. 10.1093/sysbio/syab070. 34450658 PMC9016570

[evaf051-B77] Wudarski J, Simanov D, Ustyantsev K, de Mulder K, Grelling M, Grudniewska M, Beltman F, Glazenburg L, Demircan T, Wunderer J, et al Efficient transgenesis and annotated genome sequence of the regenerative flatworm model *Macrostomum lignano*. Nat Commun. 2017:8(1):2120. 10.1038/s41467-017-02214-8. 29242515 PMC5730564

[evaf051-B78] Yi M, Chen F, Luo M, Cheng Y, Zhao H, Cheng H, Zhou R. Rapid evolution of piRNA pathway in the teleost fish: implication for an adaptation to transposon diversity. Genome Biol Evol. 2014:6(6):1393–1407. 10.1093/gbe/evu105. 24846630 PMC4079211

[evaf051-B79] Zadesenets KS, Schärer L, Rubtsov NB. New insights into the karyotype evolution of the free-living flatworm *Macrostomum lignano* (Platyhelminthes, Turbellaria). Sci Rep. 2017:7(1):6066. 10.1038/s41598-017-06498-0. 28729552 PMC5519732

[evaf051-B80] Zarowiecki M, Berriman M. What helminth genomes have taught us about parasite evolution. Parasitology. 2015:142(Suppl 1):S85–S97. 10.1017/S0031182014001449. 25482650 PMC4413821

