# Supplementary material for: The Early Evolution of Tudor Genes in Holozoa and How Their Distribution Was Influenced by Life History Traits in Metazoa
Source: Genome Biol Evol. 2025 Jun 9;17(6):evaf051. doi: 10.1093/gbe/evaf051 (PMC12147562; doi:10.1093/gbe/evaf051)
Supplement: evaf051_Supplementary_Data [file evaf051_supplementary_data.zip › Extended_references.pdf]

Extended references of Table 1

Ayyanathan, K., Lechner, M. S., Bell, P., Maul, G. G., Schultz, D. C., Yamada, Y., Tanaka, K., Torigoe, K., & Rauscher, F. J. (2003). Regulated recruitment of HP1 to a euchromatic gene induces mitotically heritable, epigenetic gene silencing: A mammalian cell culture model of gene variegation. *Genes and Development*, 17(15), 1855–1869.

Bian, C., Xu, C., Ruan, J., Lee, K. K., Burke, T. L., Tempel, W., Barsyte, D., Li, J., Wu, M., Zhou, B. O., Fleharty, B. E., Paulson, A., Allali-Hassani, A., Zhou, J. Q., Mer, G., Grant, P. A., Workman, J. L., Zang, J., & Min, J. (2011). Sgf29 binds histone H3K4me2/3 and is required for SAGA complex recruitment and histone H3 acetylation. *EMBO Journal*, 30(14), 2829–2842.

Bostick, M., Kim, J.K., Estève, P.O., Clark, A., Pradhan, S., Jacobsen, S.E. (2007). UHRF1 Plays a Role in Maintaining DNA Methylation in Mammalian Cells. *Science* 317(5845):1760-4.

Bunting, S. F., Callén, E., Wong, N., Chen, H. T., Polato, F., Gunn, A., Bothmer, A., Feldhahn, N., Fernandez-Capetillo, O., Cao, L., Xu, X., Deng, C. X., Finkel, T., Nussenzweig, M., Stark, J. M., & Nussenzweig, A. (2010). 53BP1 inhibits homologous recombination in brca1-deficient cells by blocking resection of DNA breaks. *Cell*, 141(2), 243–254.

Callen, E., di Virgilio, M., Kruhlak, M. J., Nieto-Soler, M., Wong, N., Chen, H. T., Faryabi, R. B., Polato, F., Santos, M., Starnes, L. M., Wesemann, D. R., Lee, J. E., Tubbs, A., Sleckman, B. P., Daniel, J. A., Ge, K., Alt, F. W., Fernandez-Capetillo, O., Nussenzweig, M. C., & Nussenzweig, A. (2013). 53BP1 mediates productive and mutagenic DNA repair through distinct phosphoprotein interactions. *Cell*, 153(6), 1266–1280.

Chen, C., Nott, T. J., Jin, J., & Pawson, T. (2011). Deciphering arginine methylation: Tudor tells the tale. *Nature Reviews Molecular Cell Biology* 12(10), 629–642.

Cui, G., Park, S., Badeaux, A. I., Kim, D., Lee, J., Thompson, J. R., Yan, F., Kaneko, S., Yuan, Z., Botuyan, M. V., Bedford, M. T., Cheng, J. Q., & Mer, G. (2012). PHF20 is an effector protein of p53 double lysine methylation that stabilizes and activates p53. *Nature Structural and Molecular Biology*, 19(9), 916–924.

Das, R., Schintzer, L., Vinopal, S., Roca, E.A., Sylvester, M., Oprisoreanu, A.M., Schoch, S., Bradke, F., Broemer, M. (2019). New roles for the de-ubiquitylating enzyme OTUD4 in an RNA–protein network and RNA granules. *Journal of Cell Science* 132:jcs229252.

Dong, C., Nakagawa, R., Oyama, K., Yamamoto, Y., Zhang, W., Dong, A., Li, Y., Yoshimura, Y., Kamiya, H., Nakayama, J. I., Ueda, J., & Min, J. (2020). Structural basis for histone variant h3tk27me3 recognition by phf1 and phf19. *ELife*, 9, 1–21.

Fei, Q., Shang, K., Zhang, J., Chuai, S., Kong, D., Zhou, T., Fu, S., Liang, Y., Li, C., Chen, Z., Zhao, Y., Yu, Z., Huang, Z., Hu, M., Ying, H., Chen, Z., Zhang, Y., Xing, F., Zhu, J., … Shou, J. (2015). Histone methyltransferase SETDB1 regulates liver cancer cell growth through methylation of P53. *Nature Communications*, 6, 8651.

Gong, W., Liang, Q., Tong, Y., Perrett, S., & Feng, Y. (2021). Structural Insight into Chromatin Recognition by Multiple Domains of the Tumor Suppressor RBBP1. *Journal of Molecular Biology*, 433, 167224.

Gui, B., Han, X., Zhang, Y., Liang, J., Wang, D., Xuan, C., Yu, Z., & Shang, Y. (2012). Dimerization of ZIP promotes its transcriptional repressive function and biological activity. *International Journal of Biochemistry and Cell Biology*, 44(6), 886–895.

Gutierrez-Beltran, E., Denisenko, T. v., Zhivotovsky, B., & Bozhkov, P. v. (2016). Tudor staphylococcal nuclease: Biochemistry and functions. *Cell Death and Differentiation* 23(11), 1739–1748.

Kolb, S. J., Battle, D. J., & Dreyfuss, G. (2007). Molecular functions of the SMN complex. *Journal of Child Neurology* 22(8), 990–994.

Ku, H. Y., & Lin, H. (2014). PIWI proteins and their interactors in piRNA biogenesis, germline development and gene expression. *National Science Review* 1(2), 205–218.

Labbé, R. M., Holowatyj, A., & Yang, Z.-Q. (2014). Histone lysine demethylase (KDM) subfamily 4: structures, functions and therapeutic potential. *American Journal Translationa Research* 6(1), 1-15.

Li, R., Zhang, H., Yu, W., Chen, Y., Gui, B., Liang, J., Wang, Y., Sun, L., Yang, X., Zhang, Y., Shi, L., Li, Y., & Shang, Y. (2009). ZIP: A novel transcription repressor, represses EGFR oncogene and suppresses breast carcinogenesis. *EMBO Journal*, 28(18), 2763–2776.

Liang, C. C., Zhan, B., Yoshikawa, Y., Haas, W., Gygi, S. P., & Cohn, M. A. (2015). UHRF1 Is a sensor for DNA interstrand crosslinks and recruits FANCD2 to initiate the Fanconi Anemia pathway. *Cell Reports*, 10(12), 1947–1956.

Linder, B., Plöttner, O., Kroiss, M., Hartmann, E., Laggerbauer, B., Meister, G., Keidel, E., & Fischer, U. (2008). Tdrd3 is a novel stress granule-associated protein interacting with the Fragile-X syndrome protein FMRP. *Human Molecular Genetics*, 17(20), 3236–3246.

Liokatis, S., Edlich, C., Soupsana, K., Giannios, I., Panagiotidou, P., Tripsianes, K., Sattler, M., Georgatos, S. D., & Politou, A. S. (2012). Solution structure and molecular interactions of lamin B receptor Tudor domain. *Journal of Biological Chemistry*, 287(2), 1032–1042.

Liu, K., Chen, C., Guo, Y., Lam, R., Bian, C., Xu, C., Zhao, D. Y., Jin, J., Mackenzie, F., Pawson, T., & Min, J. (2010). Structural basis for recognition of arginine methylated Piwi proteins by the extended Tudor domain. *PNAS*, 107(43), 18398–18403.

Livigni, A., Scorziello, A., Agnese, S., Adornetto, A., Carlucci, A., Garbi, C., Castaldo, I., Annunziato, L., Avvedimento, E. v, & Feliciello, A. (2006). Mitochondrial AKAP121 Links cAMP and src Signaling to Oxidative Metabolism. *Molecular Biology of the Cell*, 17, 263–271.

Mevissen, T.E.T., Hospenthal, M.K., Geurink, P.P., Elliot, P.R., Akutsu, M., Arnaudo, N., Ekkebus, R., Kulathu, Y., Wauer, T., Oualid, F.E., Freund, S.M.V., Ovaa, H., Komander, D. (2013). OTU Deubiquitinases Reveal Mechanisms of Linkage Specificity and Enable Ubiquitin Chain Restriction Analysis. Cell 154:169-84.

Nikolakaki, E., Mylonis, I., & Giannakouros, T. (2017). Lamin B receptor: Interplay between structure, function and localization. *Cells* 6(28).

Sato, K., Iwasaki, Y. W., Shibuya, A., Carninci, P., Tsuchizawa, Y., Ishizu, H., Siomi, M. C., & Siomi, H. (2015). Krimper Enforces an Antisense Bias on piRNA Pools by Binding AGO3 in the *Drosophila* Germline. *Molecular Cell*, *59*(4), 553–563.

Steinhauer, W.R., Kalfayan, L.J. (1992). A specific ovarian tumor protein isoform is required for efficient differentiation of germ cells in *Drosophila* oogenesis. *Genes and Development* 6:233-43.

Tanaka, T., Hosokawa, M., Vagin, V. v., Reuter, M., Hayashi, E., Mochizuki, A. L., Kitamura, K., Yamanaka, H., Kondoh, G., Okawa, K., Kuramochi-Miyagawa, S., Nakano, T., Sachidanandam, R., Hannon, G. J., Pillai, R. S., Nakatsuji, N., & Chuma, S. (2011). Tudor domain containing 7 (Tdrd7) is essential for dynamic ribonucleoprotein (RNP) remodeling of chromatoid bodies during spermatogenesis. *PNAS*, 108(26), 10579–10584.

Vagin, V. v., Wohlschlegel, J., Qu, J., Jonsson, Z., Huang, X., Chuma, S., Girard, A., Sachidanandam, R., Hannon, G. J., & Aravin, A. A. (2009). Proteomic analysis of murine Piwi proteins reveals a role for arginine methylation in specifying interaction with Tudor family members. *Genes and Development*, 23(15), 1749–1762.

Whetstine, J. R., Nottke, A., Lan, F., Huarte, M., Smolikov, S., Chen, Z., Spooner, E., Li, E., Zhang, G., Colaiacovo, M., & Shi, Y. (2006). Reversal of Histone Lysine Trimethylation by the JMJD2 Family of Histone Demethylases. *Cell*, 125(3), 467–481.

Yabuta, Y., Ohta, H., Abe, T., Kurimoto, K., Chuma, S., & Saitou, M. (2011). TDRD5 is required for retrotransposon silencing, chromatoid body assembly, and spermiogenesis in mice. *Journal of Cell Biology*, 192(5), 781–795.

Yuan, W., Al-Hadid, Q., Wang, Z., Shen, L., Cho, H., Wu, X., & Yang, Y. (2021). TDRD3 promotes DHX9 chromatin recruitment and R-loop resolution. *Nucleic Acids Research*, 49(15), 8573–8591.

Zamparini, A. L., Davis, M. Y., Malone, C. D., Vieira, E., Zavadil, J., Sachidanandam, R., Hannon, G. J., & Lehmann, R. (2011). Vreteno, a gonad-specific protein, is essential for germline development and primary pirna biogenesis in *Drosophila*. *Development*, 138(18), 4039–4050.

Extended references for metazoan species tree topology

Bernot, J. P. *et al.* Major Revisions in Pancrustacean Phylogeny and Evidence of Sensitivity to Taxon Sampling. *Molecular Biology and Evolution* **40**, msad175 (2023).

Cranston, P. S. & Gullan, P. J. Phylogeny of Insects. in *Encyclopedia of Insects* 780–793 (Elsevier, 2009).

Giribet, G. & Edgecombe, G. D. The Phylogeny and Evolutionary History of Arthropods. *Current Biology* **29**, R592–R602 (2019).

Giribet, G. Current views on chelicerate phylogeny—A tribute to Peter Weygoldt. *Zoologischer Anzeiger* **273**, 7–13 (2018).

Irie, N., Satoh, N. & Kuratani, S. The phylum Vertebrata: a case for zoological recognition. *Zoological Lett* **4**, 32 (2018).

Laumer, C. E. & Giribet, G. Inclusive taxon sampling suggests a single, stepwise origin of ectolecithality in Platyhelminthes. *Biological Journal of the Linnean Society* **111**, 570–588 (2014).

Riutort, M., Álvarez-Presas, M., Lázaro, E., Sol, E. & Paps, J. Evolutionary history of the Tricladida and the Platyhelminthes: an up-to-date phylogenetic and systematic account. *Int. J. Dev. Biol.* **56**, 5–17 (2012).

Satoh, N., Rokhsar, D. & Nishikawa, T. Chordate evolution and the three-phylum system. *Proc. R. Soc. B.* **281**, 20141729 (2014).

Schrödl, M. & Stöger, I. A review on deep molluscan phylogeny: old markers, integrative approaches, persistent problems. *Journal of Natural History* **48**, 2773–2804 (2014).

Sharma, P. P. Chelicerates. *Current Biology* **28**, R774–R778 (2018).

Simakov, O. *et al.* Hemichordate genomes and deuterostome origins. *Nature* **527**, 459–465 (2015).

Stöger, I. *et al.* The Continuing Debate on Deep Molluscan Phylogeny: Evidence for Serialia (Mollusca, Monoplacophora + Polyplacophora). *BioMed Research International* **2013**, e407072 (2013).

Struck, T. H. *et al.* Annelid phylogeny and the status of Sipuncula and Echiura. *BMC Evol Biol* **7**, 57 (2007).

Struck, T. H. *et al.* Phylogenomic analyses unravel annelid evolution. *Nature* **471**, 95–98 (2011).

Wanninger, A. & Wollesen, T. The evolution of molluscs. *Biological Reviews* **94**, 102–115 (2019).

Weigert, A. & Bleidorn, C. Current status of annelid phylogeny. *Org Divers Evol* **16**, 345–362 (2016).

Yeates, D. K., Meusemann, K., Trautwein, M., Wiegmann, B. & Zwick, A. Power, resolution and bias: recent advances in insect phylogeny driven by the genomic revolution. *Current Opinion in Insect Science* **13**, 16–23 (2016).
