# Supplementary material for: The Early Evolution of Tudor Genes in Holozoa and How Their Distribution Was Influenced by Life History Traits in Metazoa
Source: Genome Biol Evol. 2025 Jun 9;17(6):evaf051. doi: 10.1093/gbe/evaf051 (PMC12147562; doi:10.1093/gbe/evaf051)
Supplement: evaf051_Supplementary_Data [file evaf051_supplementary_data.zip › Supplementary_Figure_Captions.pdf]

Supplementary Figure Captions

Supplementary Figures 1 to 6. ML tree topologies of the IQTREE replicates of the Tudor domain alignment. A 50% subsample of filtered Tudor domains of all OGs (with the exclusion of the noisy OG164; see Materialsand Methods) were aligned and 10 ML tree replicates were inferred. The corresponding number of the IQTREEreplicate (referring to Supplementary Files) is stated in each figure legend (replicates 3, 7, and 9 failed topology tests). Branches are colored according to the corresponding bootstrap support, through a gradientthat goes from red (lower values) to blue (greater values), as shown by the lower legend. Two outer circles are depicted. The most internal represents the Tudor domain type for each domain sequence, while the outerone corresponds to the OG from which the sequence was collected. The colors of both circles indicate the secondary structure of the Tudor domain sequence. In green T0 Tudor domain (no N-terminal structures), inpurple T1 (one N-terminal α-helix) and in yellow T2 (with 2 β-strands and a α-helix N-t).

- **Supplementary Figure 1.** Topology of replicate 1 (referring to tree file names in Supplementary Materials)
- **Supplementary Figure 2.** Topology of replicate 2
- **Supplementary Figure 3.** Topology of replicate 5
- **Supplementary Figure 4.** Topology of replicate 6
- **Supplementary Figure 5.** Topology of replicate 8
- **Supplementary Figure 6.** Topology of replicate 10

Supplementary Figure 7. Alignment of N-t position of Piwi-like homologues in Metazoa and Ichthyosporea species. Some representative species were chosen for the alignment. In red squares are depicted the conserved RG motifs (present also in the *Ichthyophonus hoferi* Piwi-like homologue at the bottom).

Supplementary Figure 8. Specie-specific pattern of Tudor OGs presence/absence. For each species is indicated the presence (colored boxes) of at least one Tudor protein sequence within the corresponding Tudor OGs. As described in Material and Methods, Tudor OGs that contained Tudor protein sequences annotated in model species are named with model homologous nomenclature, while we kept the OrthoFinder nomenclature when model homologues were not present within the OG. The boxes are colored based on the secondary structure of the Tudor domain characterizing the respective OG. In green T0 Tudor domains (without N-t extensions), in purple T1 (with a N-terminal α-helix) and in yellow T2 (with 2 β-strands and a α-helix N-t). The species are grouped based on belonging phylum.

Supplementary Figure 9. Repeat landscape profile of *Macrostomum lignano*. The plot represents the percentage of genome occupied by transposable elements (y axis) in each bin of CpG corrected kimura divergence (x axis) between all insertions and their respective consensus sequence as a proxy of the time of insertion. Young bursts of TE activity have low divergence and are reported on the left side of the plot. TE annotation was performed via RepeatMasker in sensitive mode (-s option) and using as custom TEs databasea de-novo consensus library produced with RepeatModeler2.
