# Supplementary material for: The Early Evolution of Tudor Genes in Holozoa and How Their Distribution Was Influenced by Life History Traits in Metazoa
Source: Genome Biol Evol. 2025 Jun 9;17(6):evaf051. doi: 10.1093/gbe/evaf051 (PMC12147562; doi:10.1093/gbe/evaf051)
Supplement: evaf051_Supplementary_Data [file evaf051_supplementary_data.zip › Supplementary_Tables.pdf]

Supplementary Tables

Supplementary Table 1. Species in the data set and accession codes of, or links to, genome assemblies. Species are ordered alphabetically by phylum. Unicellular Holozoa are at the bottom of the Table (the last 4 clades).

Phylum	Species	Genome code
**Annelida**		
	Capitella teleta	GCA_000328365.1
	Dimorphilus gyrociliatus	GCA_904063045.1
	Helobdella robusta	GCF_000326865.1
	Owenia fusiformis	GCA_903813345.1
**Arthropoda**		
	Aphis gossypii	GCF_004010815.1
	Apis mellifera	GCF_003254395.2
	Centruroides sculpturatus	GCF_000671375.1
	Cloeon dipterum	GCA_902829235.1
	Cryptotermes secundus	GCF_002891405.2
	Ctenocephalides felis	GCF_003426905.1
	Daphnia magna	GCF_003990815.1
	Darwinula stevensoni	GCA_905338385.1
	Dermatophagoides pteronyssinus	GCF_001901225.1
	Drosophila melanogaster	GCF_000001215.4
	Eurytemora affinis	GCF_000591075.1
	Folsomia candida	GCF_002217175.1
	Hyalella azteca	GCF_000764305.1
	Ixodes scapularis	GCF_002892825.2
	Lepeophtheirus salmonis	GCF_016086655.3
	Limulus polyphemus	GCF_000517525.1
	Nymphon striatum	GCA_016618385.1
	Parasteatoda tepidariorum	GCF_000365465.2
	Penaeus vannamei	GCF_003789085.1
	Sitophilus oryzae	GCF_002938485.1
	Tetranychus urticae	GCF_000239435.1
	Varroa destructor	GCF_002443255.1
**Brachiopoda**		
	Lingula anatina	GCF_001039355.2
**Bryozoa**		
	Bugula neritina	GCA_010799875.2
**Chordata**		
	Acipenser ruthenus	GCF_010645085.1
	Amblyraja radiata	GCF_010909765.1
	Branchiostoma floridae	GCF_000003815.2
	Ciona intestinalis	GCF_000224145.3
	Danio rerio	GCF_000002035.6
	Gallus gallus	GCF_000002315.6
	Gekko japonicus	GCF_001447785.1
	Homo sapiens	GCF_000001405.39
	Oikopleura dioica	GCA_000209555.1
	Petromyzon marinus	GCF_010993605.1
	Styela clava	GCF_013122585.1
	Xenopus tropicalis	GCF_000004195.4
**Cnidaria**		
	Acropora digitifera	GCF_000222465.1
	Actinia tenebrosa	GCF_009602425.1
	Aurelia aurita	https://marinegenomics.oist.jp/aurelia_aurita/viewer/info?project_id=69
	Clytia hemisphaerica	http://ftp.ensemblgenomes.org/pub/metazoa/release-52/fasta/clytia_hemisphaerica_gca902728285/pep/
	Dendronephthya gigantea	GCF_004324835.1
	Exaiptasia diaphana	GCF_001417965.1
	Henneguya salminicola	GCA_009887335.1
	Hydra vulgaris	GCF_000004095.1
	Morbakka virulenta	https://marinegenomics.oist.jp/morba

		kka_virulenta/viewer/info?project_id=70
	Myxobolus squamalis	GCA_010108815.1
	Nematostella vectensis	GCF_000209225.1
	Orbicella faveolata	GCF_002042975.1
	Pocillopora damicornis	GCF_003704095.1
	Stylophora pistillata	GCF_002571385.1
	Thelohanellus kitauei	GCA_000827895.1
**Ctenophora**		
	Hormiphora californiensis	https://github.com/conchoecia/hormiphora/blob/master/annotation/proteins/h1flnc_to_Jan24_annoth1.pilon.fasta.transdecoder_dir/h1_longest_orfs.pep.gz
	Mnemiopsis leidyi	https://research.nhgri.nih.gov/mnemiopsis/
**Echinodermata**		
	Anneissia japonica	GCF_011630105.1
	Apostichopus japonicus	GCA_002754855.1
	Asterias rubens	GCF_902459465.1
	Strongylocentrotus purpuratus	GCF_000002235.5
**Hemichordata**		
	Ptychodera flava	https://marinegenomics.oist.jp/acornworm/viewer/info?project_id=33
	Saccoglossus kowalevskii	GCF_000003605.2
**Mollusca**		
	Aplysia californica	GCF_000002075.1
	Biomphalaria glabrata	GCF_000457365.1
	Crassostrea gigas	GCF_902806645.1
	Lottia gigantea	GCF_000327385.1
	Mizuhopecten yessoensis	GCF_002113885.1
	Octopus bimaculoides	GCF_001194135.1
	Pomacea canaliculata	GCF_003073045.1
**Nematoda**		
	Aphelenchus avenae	GCA_020875895.1
	Brugia malayi	GCF_000002995.3
	Bursaphelenchus okinawaensis	GCA_904066225.2
	Caenorhabditis elegans	GCF_000002985.6
	Loa loa	GCF_000183805.1
	Necator americanus	GCF_000507365.1
	Plectus sambesii	https://parasite.wormbase.org/Plectus_sambesii_prjna390260/Info/Index/
	Strongyloides ratti	GCF_001040885.1
	Trichinella spiralis	GCF_000181795.1
**Orthonectida**		
	Intoshia linei	GCA_001642005.1
**Phoronida**		
	Phoronis australis	https://marinegenomics.oist.jp/pau_v2/viewer/info?project_id=51
**Placozoa**		
	Trichoplax adhaerens	GCF_000150275.1
	Trichoplax spH2	GCF_000150275.1
**Platyhelminthes**		
	Dibothriocephalus latus	GCA_900617775.1
	Echinococcus granulosus	GCF_000524195.1
	Fasciola hepatica	GCA_002763495.2
	Macrostomum lignano	GCA_002269645.1
	Opisthorchis viverrini	GCF_000715545.1
	Protopolystoma xenopodis	GCA_900617795.1
	Schistosoma mansoni	GCF_000237925.1
	Schmidtea mediterranea	https://planmine.mpibpc.mpg.de
**Porifera**		
	Amphimedon queenslandica	GCF_000090795.1
	Ephydatia muelleri	https://spaces.facsci.ualberta.ca/ephybase/
**Priapulida**		
	Priapulus caudatus	GCF_000485595.1
**Rotifera**		
	Adineta ricciae	GCA_905250095.1

	Brachionus calyciflorus	GCA_905250105.1
	Didymodactylos carnosus	GCA_905250885.1
	Rotaria socialis	GCA_905332285.1
**Tardigrada**		
	Hypsibius dujardini	GCA_002082055.1
	Ramazzottius varieornatus	GCA_001949185.1
**Xenacoelomorpha**		
	Praesagittifera naikaiensis	http://gigadb.org/dataset/100564
	Xenoturbella bocki	https://figshare.com/articles/dataset/Genome_of_Xenoturbella_bocki/7679948?file=14279699
**Choanoflagellata**		
	Monosiga brevicollis	http://ftp.ensemblgenomes.org/pub/protists/release-52/fasta/protists_choanoflagellida1_collection/monosiga_brevicollis_mx1_gca_000002865/pep/
	Salpingoeca rosetta	GCA_000188695.1
**Ichthyosporea**		
	Chromosphaera perkinsii	https://figshare.com/articles/dataset/Genome_-_Chromosphaera_perkinsii/5426494?file=9362518
	Ichthyophonus hoferi	https://figshare.com/articles/dataset/Genome_-_Ichthyophonus_hoferi/5426488?file=9362497
	Pirum gemmata	https://figshare.com/articles/dataset/Genome_-_Pirum_gemmata/5426506?file=10442961
	Sphaeroforma arctica	GCF_001186125.1
**Pluriformea**		
	Corallochytrium limacisporum	https://figshare.com/articles/dataset/Genome_-_Corallochytrium_limacisporum/5426470?file=9362476
**Filasterea**		
	Capsaspora owczarzaki	GCF_000151315.2

Supplementary Table 2. Species-specific genomic and genetic statistics used for statistical analyses. Species are in alphabetical order. Unicellular Holozoa are at the bottom of the table

Species	Genome size (Mb)	Gene Number	Gene density	Splicing index	T0	T1	T3	Piwi-like	Ago-like	BUSCO v5 (Metazoa) completeness
Acipenser ruthenus	1828.86	36150	19.77	0.54	40	10	22	4	8	98.2
Acropora digitifera	449.88	26073	57.96	0.77	11	5	21	3	5	77.5
Actinia tenebrosa	234.72	19980	85.12	0.74	18	3	17	3	2	94
Adineta ricciae	176.04	64538	366.61	NA	35	8	59	9	35	87.8
Amblyraja radiata	2562.36	18512	7.22	0.48	19	4	14	3	4	95.9
Amphimedon queenslandica	166.26	20788	125.03	0.86	8	1	17	2	2	90.8
Anneissia japonica	586.8	21084	35.93	0.64	13	3	14	2	3	95.1
Aphelenchus avenae	264.06	43185	163.54	NA	12	3	16	0	23	74
Aphis gossypii	293.4	12828	43.72	0.69	13	2	22	6	5	93.3
Apis mellifera	224.94	9935	44.17	0.42	12	3	10	2	2	98.7
Aplysia californica	929.1	19405	20.89	0.73	12	2	18	2	2	91.4
Apostichopus japonicus	801.96	30221	37.68	NA	18	4	20	3	2	84.3

Asterias rubens	420.54	16079	38.23	0.67	13	3	15	2	1	98.3
Aurelia aurita	381.42	28604	74.99	0.75	11	2	20	2	3	74.8
Biomphalaria glabrata	919.32	25552	27.79	0.7	11	2	21	2	1	88.6
Brachionus calyciflorus	117.36	24328	207.29	NA	7	3	22	4	3	90.1
Branchiostoma floridae	518.34	26689	51.49	0.62	11	4	18	6	2	95.5
Brugia malayi	97.8	11371	116.27	0.99	9	4	17	0	6	75.1
Bugula neritina	215.16	25318	117.67	NA	14	4	20	2	3	76.3
Bursaphelenchus okinawaensis	68.46	14593	213.16	NA	5	2	7	1	4	74.8
Caenorhabditis elegans	97.8	21903	223.96	0.72	7	2	8	1	9	77.9
Capitella teleta	332.52	31978	96.17	NA	13	3	13	3	1	94.6
Centruroides sculpturatus	929.1	24591	26.47	0.69	15	3	24	4	23	93.4
Ciona intestinalis	117.36	13713	116.85	0.65	11	3	9	2	1	93.1
Cloeon dipterum	176.04	30161	171.33	NA	22	4	39	12	10	95.9
Clytia hemisphaerica	449.88	19149	42.56	0.74	13	2	19	2	2	77.9
Crassostrea gigas	645.48	31371	48.6	0.5	11	4	19	2	3	98.2
Cryptotermes secundus	1017.12	13170	12.95	0.45	10	3	12	3	12	97.6
Ctenocephalides felis	772.62	18878	24.43	0.86	17	3	21	5	2	96.8
Danio rerio	1369.2	26533	19.38	0.46	23	4	15	2	5	98
Daphnia magna	127.14	15351	120.74	0.65	13	3	19	6	3	95.9
Darwinula stevensoni	382.1	15746	41,21	NA	8	3	15	3	2	78
Dendronephthya gigantea	283.62	22045	77.73	0.77	12	3	19	1	4	94.3
Dermatophagoides pteronyssinus	68.46	11184	163.37	0.87	8	2	10	0	8	87.2
Dibothriocephalus latus	528.12	19966	37.81	NA	5	2	1	0	5	32.9
Dicyema japonicum	68.46	4743	69.28	0.95	0	1	2	4	0	32.3
Didymodactylos carnosus	371.64	46863	126.1	NA	16	7	30	8	20	81.3
Dimorphilus gyrociliatus	78.24	16378	209.33	NA	9	4	15	2	1	91.8
Drosophila melanogaster	146.7	13955	95.13	0.45	8	4	16	3	2	99.3
Echinococcus granulosus	107.58	11319	105.22	1	6	2	2	0	3	68.4
Ephydatia muelleri	322.74	39329	121.86	NA	8	1	28	2	0	69.7
Eurytemora affinis	391.2	20716	52.96	0.68	13	3	27	4	5	87.8
Exaiptasia diaphana	254.28	22509	88.52	0.81	16	3	18	4	3	91.8
Fasciola hepatica	1134.48	11217	9.89	NA	5	2	2	0	4	62.4
Folsomia candida	224.94	24221	107.68	0.65	11	3	45	19	11	94.3
Gallus gallus	1066.02	17576	16.49	0.35	20	4	11	2	3	94.2
Gekko japonicus	2493.9	19548	7.84	0.8	20	4	14	3	4	94.7
Helobdella robusta	234.72	23426	99.8	1	11	2	14	2	2	90.2
Henneguya salminicola	58.68	8187	139.52	NA	1	2	4	2	2	26.8
Homo sapiens	3100.26	20331	6.56	0.17	22	5	13	4	4	99.1
Hormiphora californiensis	97.8	11987	122.57	0.68	8	2	16	2	3	74.1
Hyalella azteca	547.68	18608	33.98	0.82	10	2	11	6	4	90.5
Hydra vulgaris	850.86	20055	23.57	0.91	11	1	17	3	2	82.2
Hypsibius dujardini	107.58	20860	193.9	NA	8	1	16	5	4	84.3
Intoshia linei	39.12	8724	223.01	NA	4	2	3	1	3	49.9
Ixodes scapularis	2083.14	24501	11.76	0.75	13	4	22	4	5	95.7
Lepeophtheirus salmonis	645.48	14014	21.71	0.68	11	4	13	4	5	92
Limulus polyphemus	1828.86	22873	12.51	0.59	19	3	20	2	3	92.3

Lingula anatina	410.76	27068	65.9	0.65	16	5	25	5	2	98.2
Loa loa	88.02	16281	184.97	NA	8	2	17	0	5	72.7
Lottia gigantea	361.86	23827	65.85	1	10	3	15	2	1	96.5
Macrostomum lignano	762.84	49018	64.26	NA	22	9	66	14	12	86.7
Mizuhopecten yessoensis	987.78	24532	24.84	0.59	13	3	15	2	1	98.3
Mnemiopsis leidyi	146.7	16548	112.8	NA	13	1	19	4	4	73.7
Morbakka virulenta	948.66	24278	25.59	0.84	11	2	20	2	1	84.4
Myxobolus squamalis	39.12	5723	146.29	NA	1	2	2	2	2	17.1
Necator americanus	244.5	19153	78.34	1	6	2	6	2	9	57.9
Nematostella vectensis	352.08	23845	67.73	0.69	20	4	24	5	3	94.9
Notospermus geniculatus	860.64	43294	50.3	1	19	6	30	7	4	93.4
Nymphon striatum	743.28	10384	13.97	0.36	13	2	8	2	3	60.2
Octopus bimaculoides	2337.42	15842	6.78	0.66	11	3	16	3	3	94.2
Oikopleura dioica	48.9	13527	276.63	NA	3	2	7	1	13	37
Opisthorchis viverrini	616.14	16356	26.55	1	8	1	2	0	4	64.2
Orbicella faveolata	489	25929	53.02	0.8	19	3	20	2	7	87.1
Owenia fusiformis	498.78	31127	62.41	0.9	12	3	19	3	1	92.5
Parasteatoda tepidariorum	1447.44	18601	12.85	0.68	16	3	14	2	8	89.7
Penaeus vannamei	1662.6	24987	15.03	0.75	10	3	12	1	3	88.6
Petromyzon marinus	1085.58	17580	16.19	0.47	15	4	14	2	3	94.6
Phoronis australis	498.78	20473	41.05	1	12	4	18	3	1	96.3
Plectus sambesii	185.82	40530	218.11	1	19	3	50	2	12	85.2
Pocillopora damicornis	234.72	19935	84.93	0.79	13	3	18	2	2	89.6
Pomacea canaliculata	440.1	21144	48.04	0.52	11	3	17	2	1	98.1
Praesagittifera naikaiensis	655.26	22143	33.79	0.91	11	1	23	3	4	67.2
Priapulus caudatus	508.56	15101	29.69	0.73	10	4	18	2	2	87.2
Protopolystoma xenopodis	616.14	37906	61.52	NA	3	0	0	0	8	21
Ptychodera flava	997.56	34637	34.72	1	10	1	22	3	1	55.9
Ramazzottius varieornatus	58.68	19533	332.87	0.85	6	1	13	5	8	85
Rotaria socialis	156.48	34499	220.47	NA	16	5	33	8	18	86.8
Saccoglossus kowalevskii	772.62	20935	27.1	0.95	9	4	17	4	1	89.7
Schistosoma mansoni	361.86	10719	29.62	0.91	8	2	2	0	3	70.9
Schmidtea mediterranea	704.16	22090	31.37	0.72	7	1	15	3	3	77.6
Sitophilus oryzae	772.62	15057	19.49	0.64	10	3	15	2	2	98.8
Strongylocentrotus purpuratus	919.32	27435	29.84	0.71	14	6	20	3	2	99.3
Strongyloides ratti	39.12	12445	318.12	1	2	0	9	0	6	68.8
Styela clava	342.3	19953	58.29	0.73	12	2	12	2	1	93.5
Stylophora pistillata	400.98	24846	61.96	0.75	13	3	15	2	6	91.8
Tetranychus urticae	88.02	11686	132.77	0.75	11	2	21	7	10	86.7
Thelohanellus kitauei	146.7	15020	102.39	NA	2	0	3	1	2	25.4
Trichinella spiralis	58.68	16380	279.14	1	1	2	12	0	47	60.6
Trichoplax adhaerens	107.58	11518	107.07	1	8	1	5	0	1	89.4
Trichoplax spH2	97.8	12174	124.48	NA	12	1	5	0	4	94.8
Varroa destructor	371.64	10260	27.61	0.34	6	3	12	0	10	94.7
Xenopus tropicalis	1447.44	21898	15.13	0.48	22	5	14	4	4	98.9
Xenoturbella bocki	537.9	24134	44.87	NA	6	1	23	5	4	46

Capsaspora owczarzaki	29.34	8792	299.66	NA	9	2	1	0	0	74.8
Corallochytrium limacisporum	244.5	7535	30.82	NA	4	0	1	0	0	59.9
Chromosphaera perkinsii	39.12	12463	318.58	NA	3	1	2	1	1	75.2
Ichthyophonus hoferi	88.02	6351	72.15	NA	6	1	2	1	2	38.7
Monosiga brevicollis	39.12	9172	234.46	NA	5	2	1	0	0	59.3
Pirum gemmata	88.02	21835	248.07	NA	2	0	1	0	2	2.6
Salpingoeca rosetta	58.68	11618	197.99	0.99	7	2	1	0	0	65.9
Sphaeroforma arctica	117.36	18213	155.19	0.97	2	2	1	0	3	47.2

Supplementary Table 3. Correlation results between Phylogenetic Independent Contrasts (PICs) for the number of the different sets of Tudor genes against some genomic features. PIC values were obtained with *pic* function of *ape* package in R. P-values were corrected with *p.adjust* in R (*fdr* method). Significant correlations (after p-value correction) are highlighted in orange. Tot: total number of genes; GeneD: Gene density; Splicing: splicing index.

Correlation	ρ	p-value	Corrected p-value
**T0vsTot**	0.27549	0.012802	0.089564
**T0vsCvalue**	0.2555078	0.021325	0.089564
**T0vsGeneD**	0.2643915	0.017072	0.089564
**T0vsSplicing**	0.1445914	0.24305	0.392613
**T0vsT1**	0.1581259	0.15856	0.302722
**T0vsT3**	0.2287245	0.039991	0.119974
**T0vsPiwi**	0.1222612	0.27688	0.415333
**T0vsAgo**	0.05150487	0.64792	0.755916
**T1vsTot**	0.0306059	0.78621	0.825520
**T1vsCvalue**	0.180906	0.106048	0.227861
**T1vsGeneD**	0.2385479	0.03198269	0.111939
**T1vsSplicing**	0.2378062	0.052648	0.138201
**T1vsT3**	0.1105827	0.325708	0.455992
**T1vsPiwi**	0.08609374	0.444735	0.583715
**T1vsAgo**	0.005286226	0.962643	0.9626432
**T3vsTot**	0.5449542	1.44158E-07	3.027328E-06
**T3vsCvalue**	0.1327143	0.237564	0.392613
**T3vsGeneD**	0.07331763	0.515382	0.636649
**T3vsSplicing**	0.1978551	0.108505	0.227861
**T3vsPiwi**	0.4287882	6,50111E-05	6.8261676E-04
**T3vsAgo**	0.03350637	0.766501	0.825520

Supplementary Table 4. Ancestral State Reconstruction (ASR) results for the 7 considered ML replicates. ASR model is the better-fitting model, by AICc, inferred by *fitDiscrete* function (ARD: a different rate for every transition; SYM: transition rates are symmetrical, e.g. T0toT1 = T1toT0; ER: one single rate for all transitions). The transition rates and the ancestral state were inferred with *corHMM* fuction.

	lnL	ASR model	Ancestral state	T0 to T1	T0 to T2	T1 to T0	T1 to T2	T2 to T1	T2 to T1
**Rep1**	-82344.0	ARD	T0	0.0074638	1E-09	0.0570717	0.0164607	0.012799	1E-09

**Rep2**	-82345.3	ARD	T0	0.0056905	0.0023662	0.0596426	1E-09	0.0095341	1E-09
**Rep4**	-82295.6	SYM	T0	0.0139912	0.0080643	0.0139912	1E-09	0.0080643	1E-09
**Rep5**	-82270.5	ER	T0	0.0055110	0.0055110	0.0055110	0.0055110	0.0055110	0.0055110
**Rep6**	-82337.0	ER	T0	0.0070611	0.0070611	0.0070611	0.0070611	0.0070611	0.0070611
**Rep8**	-82395.3	ARD	T0	0.0025043	0.0018371	0.0529876	1E-09	1E-09	0.0049324
**Rep10**	-82389.5	ARD	T0	0.0042795	0.0026670	0.1199331	1E-09	0.0217133	0.0046411

Supplementary Table 5. Diamond-BLASTP results of choanoflagellate T3+DCD proteins (from Bell et al. 2020) against the proteomes of our unicellular holozoan species. The only Diamond (*--ultra-sensitive --e-value 1e-05*) hits that we could find for the T3+DCD proteins against our unicellular holozoan proteomes were sequences homologous to Phosphorylated CTD-interacting factor 1 (PCIF1) of *H. sapiens* and *D. melanogaster*, driven by similarities between CAPAM-like MTase domain (of the T3+DCD proteins) and PCIF1 WW domain (of PCIF1). Score = BLASTP bitscore; %I = percentage of identity; Len = length of alignment; St = Starting position on subject sequence (Protein of present study Holozoa); En = Ending position on subject sequence

Choanoflagellata (Bell et al.)	Protein Identifier of Present Study Specie	Score	%I	Len	St	En
09_Salpingoeca_kvevrii	Sphaeroforma_arctica_NW_014040122.1_prot_XP_014155351.1_6446	118.2	33.1	338	235	539
09_Salpingoeca_kvevrii	Sphaeroforma_arctica_NW_014040555.1_prot_XP_014161664.1_138	115.2	33.9	233	297	498
09_Salpingoeca_kvevrii	Salpingoeca_rosetta_NW_004754917.1_prot_XP_004992160.1_6818	90.5	32.0	222	79	274
09_Salpingoeca_kvevrii	Sphaeroforma_arctica_NW_014040451.1_prot_XP_014159319.1_2503	92.8	43.2	118	449	564
09_Salpingoeca_kvevrii	Monosiga_brevicollis_EDQ90865	79.7	32.9	173	382	551
09_Salpingoeca_kvevrii	Salpingoeca_rosetta_NW_004754896.1_prot_XP_004988087.1_11000	65.5	36.8	106	694	799
09_Salpingoeca_kvevrii	Pirum_gemmata_Pgem_evm1s10301	55.1	46.9	64	77	138
04_Salpingoeca_urceolata	Sphaeroforma_arctica_NW_014040555.1_prot_XP_014161664.1_138	132.1	37.8	222	305	496
04_Salpingoeca_urceolata	Sphaeroforma_arctica_NW_014040122.1_prot_XP_014155351.1_6446	116.3	33.0	276	287	545
04_Salpingoeca_urceolata	Salpingoeca_rosetta_NW_004754917.1_prot_XP_004992160.1_6818	105.1	30.7	218	79	269
04_Salpingoeca_urceolata	Sphaeroforma_arctica_NW_014040451.1_prot_XP_014159319.1_2503	102.8	30.8	224	437	629
04_Salpingoeca_urceolata	Monosiga_brevicollis_EDQ90865	97.8	31.4	274	389	633
04_Salpingoeca_urceolata	Salpingoeca_rosetta_NW_004754896.1_prot_XP_004988087.1_11000	78.6	29.2	243	668	874
04_Salpingoeca_urceolata	Pirum_gemmata_Pgem_evm1s10301	55.8	44.9	69	72	138

Supplementary Table 6. Independent origins of endoparasitism. Independent origins are based on Weinstein and Kuris 2016. Species within the same quadrant share a supposedly common origin of endoparasitism.

Phylum	Species
**Cnidaria (Myxozoa)**	*Myxobolus squamalis*<br>*Thelohanellus kitauei*<br>*Henneguya salminicola*
**Dicyiemida**	*Dicyema japonicum*
**Orthonectida**	*Intoshia linei*
**Platyhelminthes (Trematoda)**	*Schistosoma mansoni*<br>*Opisthorchis viverrine*<br>*Echinococcus granulosus*<br>*Fasciola hepatica*<br>*Protopolystoma xenopodis*
**Nematoda**	*Loa loa*

	Brugia malayi
	Trichinella spiralis
	Necator americanus
	Strongyloides ratti

Supplementary Table 7. InterProScan codes (IPR codes) used to annotate the OGs for Tudor domain-containing proteins

Accession	Name	Type	Integrated into
IPR002999	Tudor domain	domain	SM00333,PF00567,PS50304,cd04508
IPR010750	SGF29 tudor-like domain	domain	PS51518,PF07039
IPR015125	Tumour suppressor p53-binding protein-1 Tudor domain	domain	PF09038
IPR021331	Hypervirulence associated protein, TUDOR domain	domain	PF11160
IPR021991	UHRF1, tandem tudor domain	domain	PF12148
IPR025963	FlgD Tudor-like domain	domain	PF13861
IPR040477	Lysine-specific demethylase 4, Tudor domain	domain	PF18104
IPR040765	RapA, N-terminal Tudor like domain 1	domain	PF18339
IPR040766	RapA, N-terminal Tudor-like domain 2	domain	PF18337
IPR041291	Histone methyltransferase, Tudor domain 1	domain	PF18359
IPR041292	Histone methyltransferase, Tudor domain 2	domain	PF18358
IPR041297	DNA repair protein Crb2, Tudor domain	domain	PF18115
IPR041560	Fxr1, tudor domain	domain	PF18336

Supplementary Table 8. Symfrac thresholds used for the different HMM profile of the OGs

OG	symfrac	OG	symfrac
**ARID4A-4B**	0.7	**TDRD9**	0.5
**FMR**	0.7	**TP53BP1**	0.5
**KDM4A-4B-4C**	0.5	**UHRF1**	0.5
**LBR**	0.5	**ZGPAT**	0.5
**OG164**	0.5	**OG0002153**	0.5
**OTU**	0.3	**OG0009299**	0.5
**PHF1-19-MTF2**	0.5	**OG0009555**	0.5
**PHF20-20L1**	0.3	**OG0010217**	0.3
**SETDB1**	0.3	**OG0013779**	0.3
**SGF29**	0.5	**OG0014308**	0.5
**SMN1-2-DC1**	0.5	**OG0015066**	0.3
**SND1**	0.5	**OG0015713**	0.7
**STK31**	0.5	**OG0018118**	0.5
**TDRD10**	0.5	**OG0019490**	0.5
**TDRD12**	0.3	**OG0019651**	0.5
**TDRD3**	0.7	**OG0019657**	0.5
