# Supplementary figures and images for: The Early Evolution of Tudor Genes in Holozoa and How Their Distribution Was Influenced by Life History Traits in Metazoa
Source: Genome Biol Evol. 2025 Jun 9;17(6):evaf051. doi: 10.1093/gbe/evaf051 (PMC12147562; doi:10.1093/gbe/evaf051)

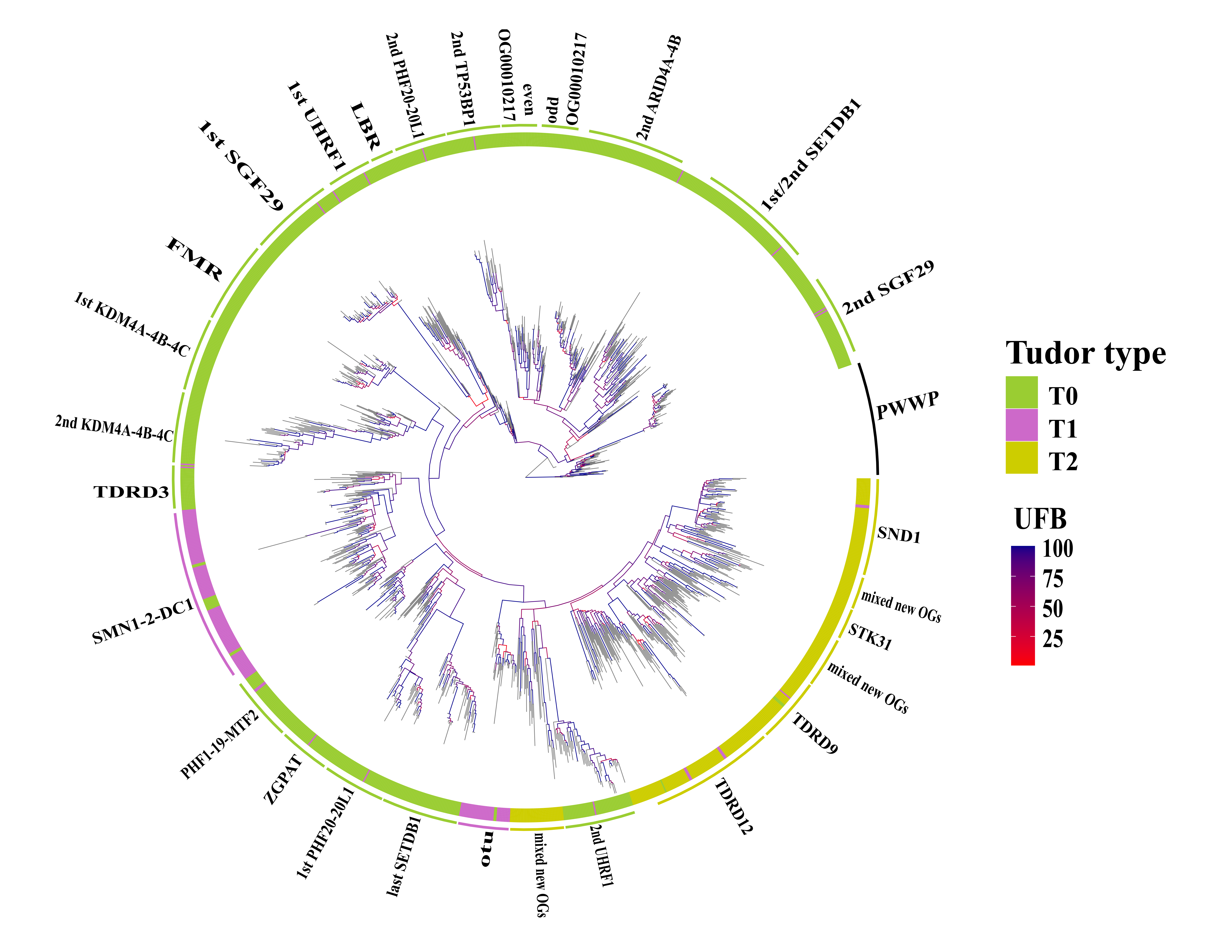

Supplement: evaf051_Supplementary_Data [file evaf051_supplementary_data.zip › Supplementary_Figure_1.png]

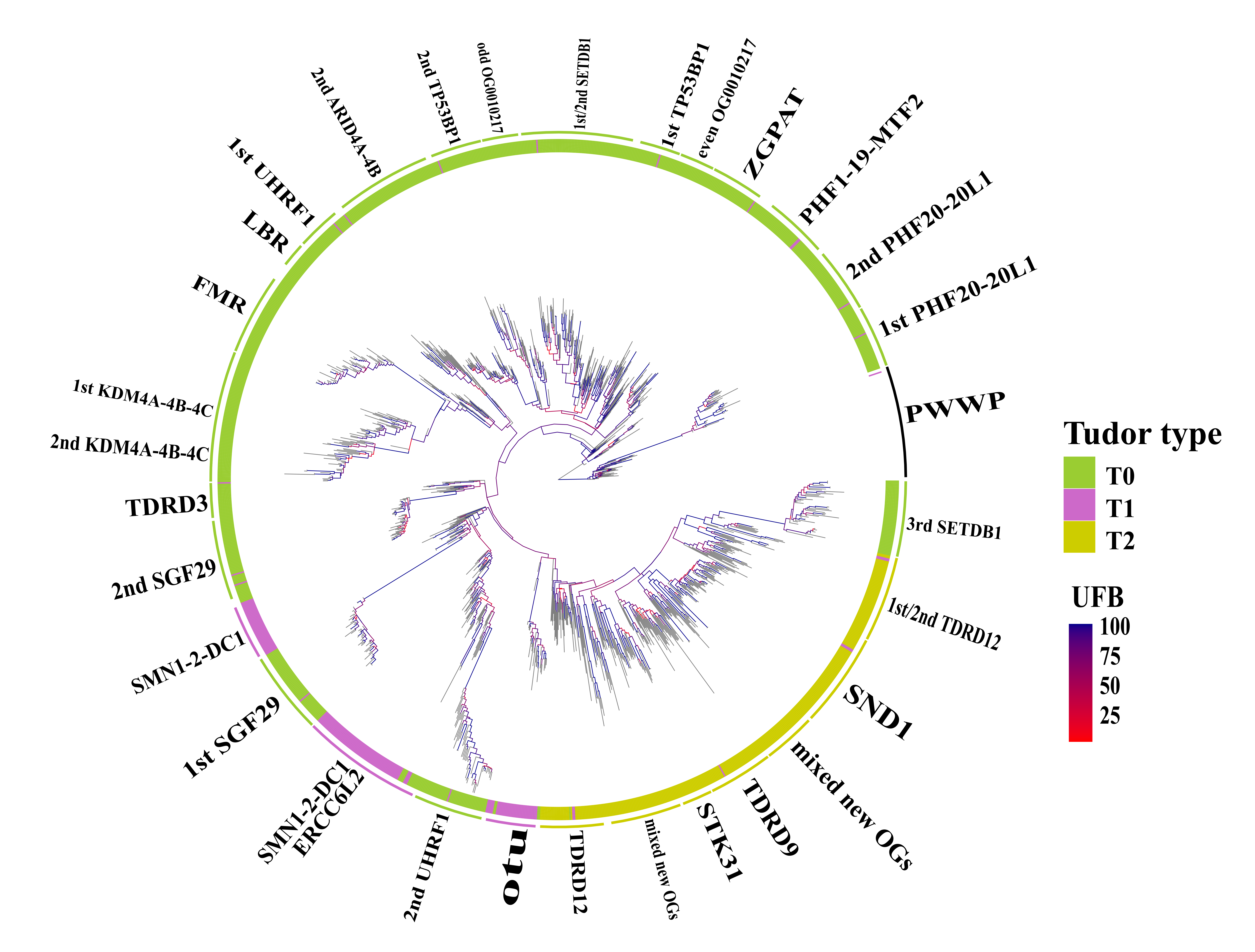

Supplement: evaf051_Supplementary_Data [file evaf051_supplementary_data.zip › Supplementary_Figure_2.png]

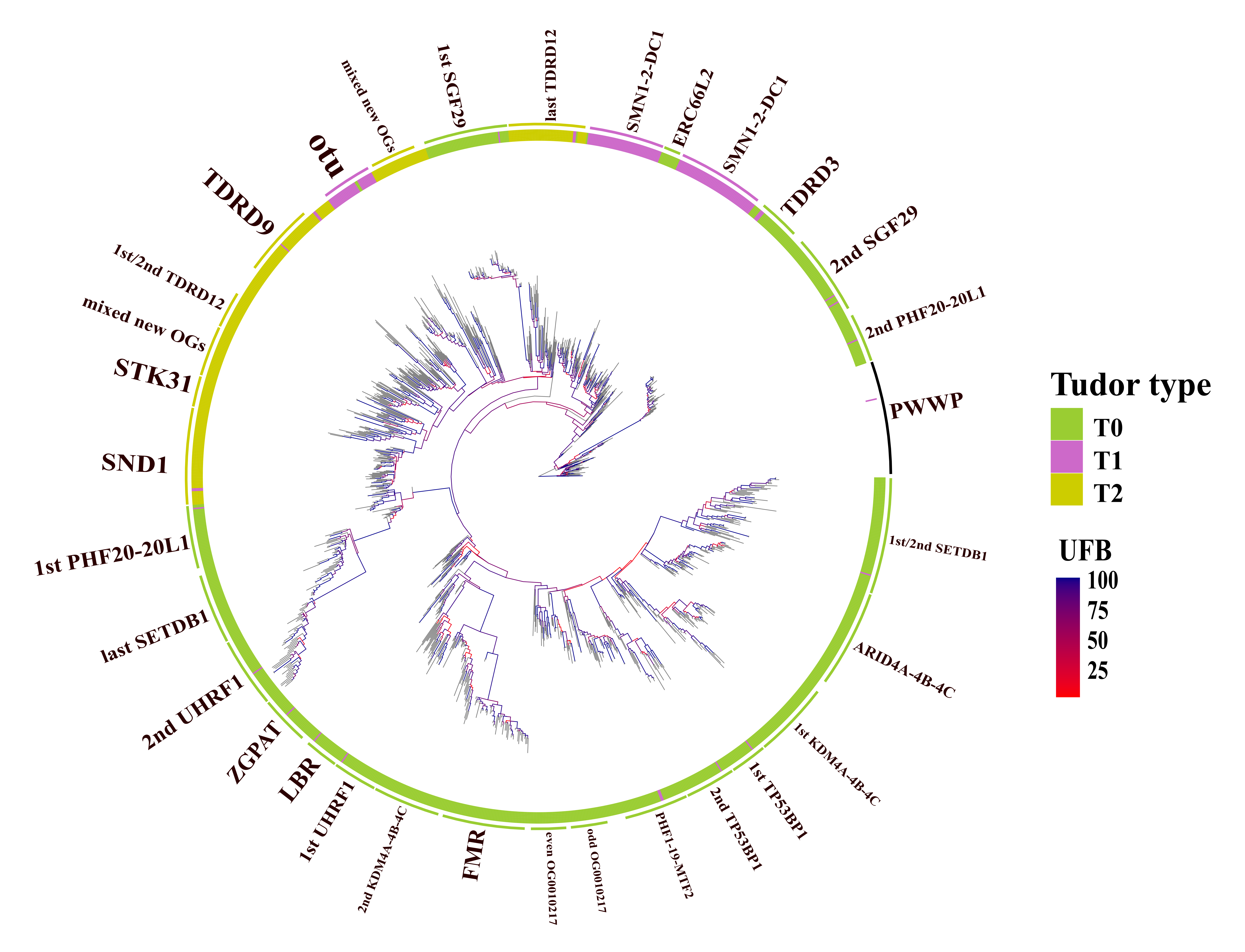

Supplement: evaf051_Supplementary_Data [file evaf051_supplementary_data.zip › Supplementary_Figure_3.png]

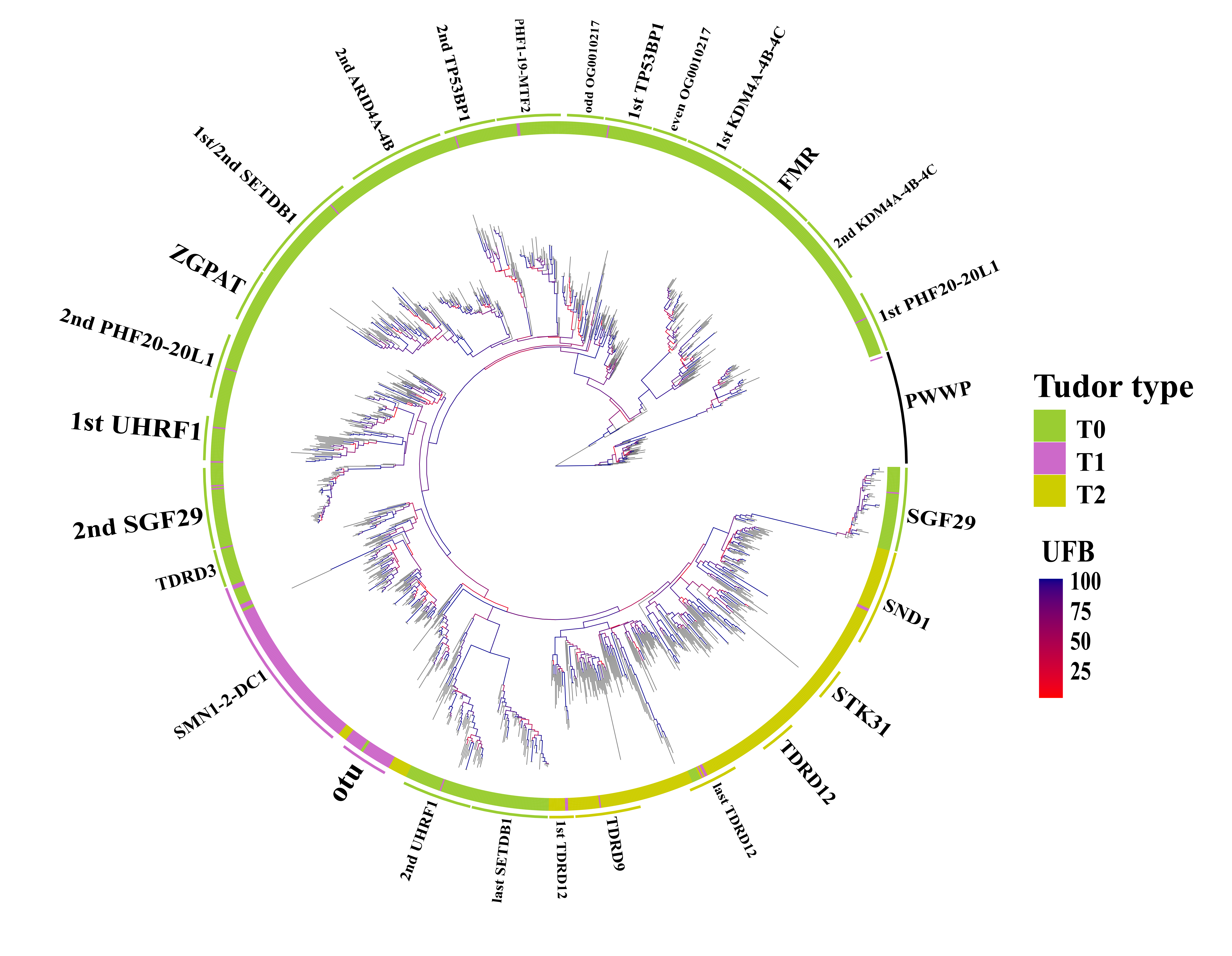

Supplement: evaf051_Supplementary_Data [file evaf051_supplementary_data.zip › Supplementary_Figure_4.png]

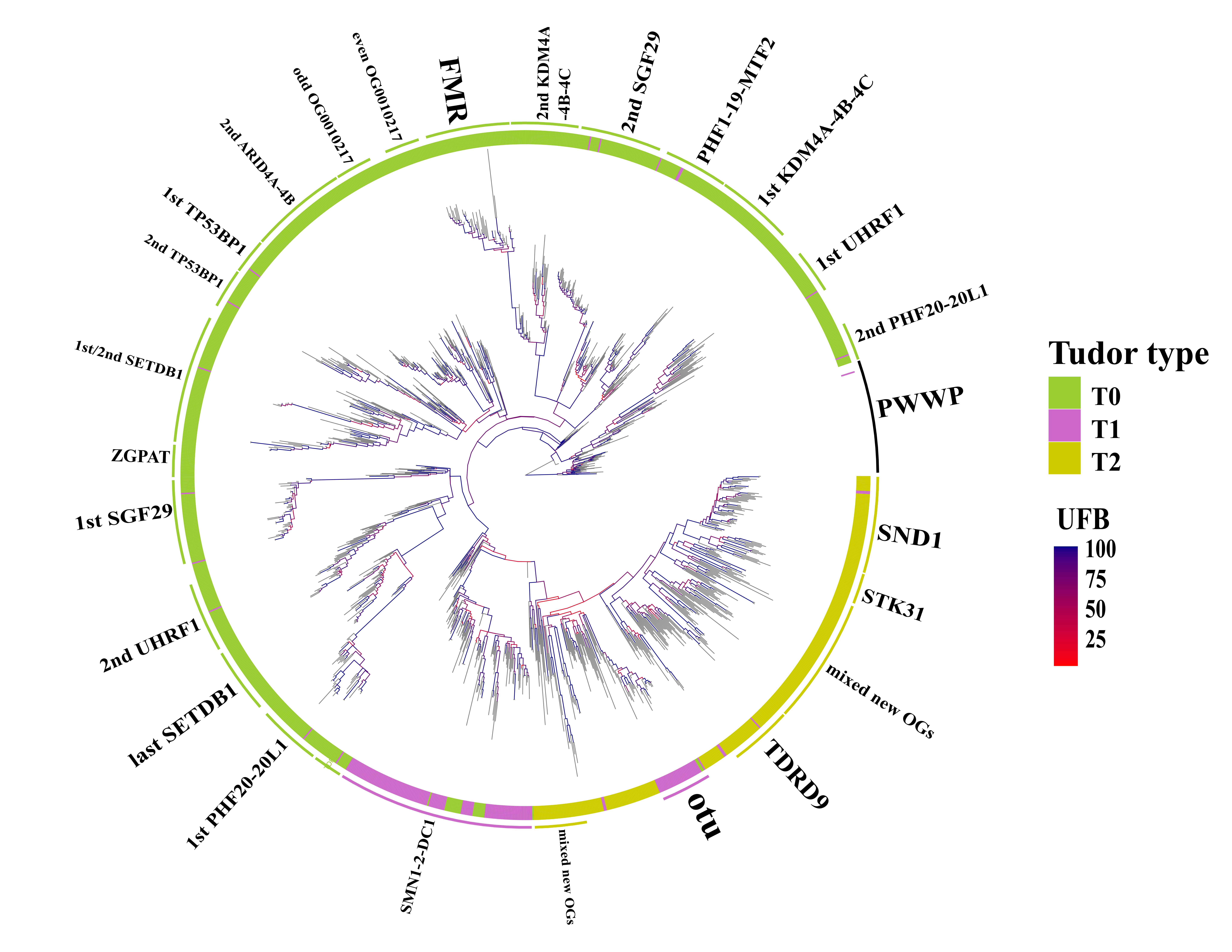

Supplement: evaf051_Supplementary_Data [file evaf051_supplementary_data.zip › Supplementary_Figure_5.png]

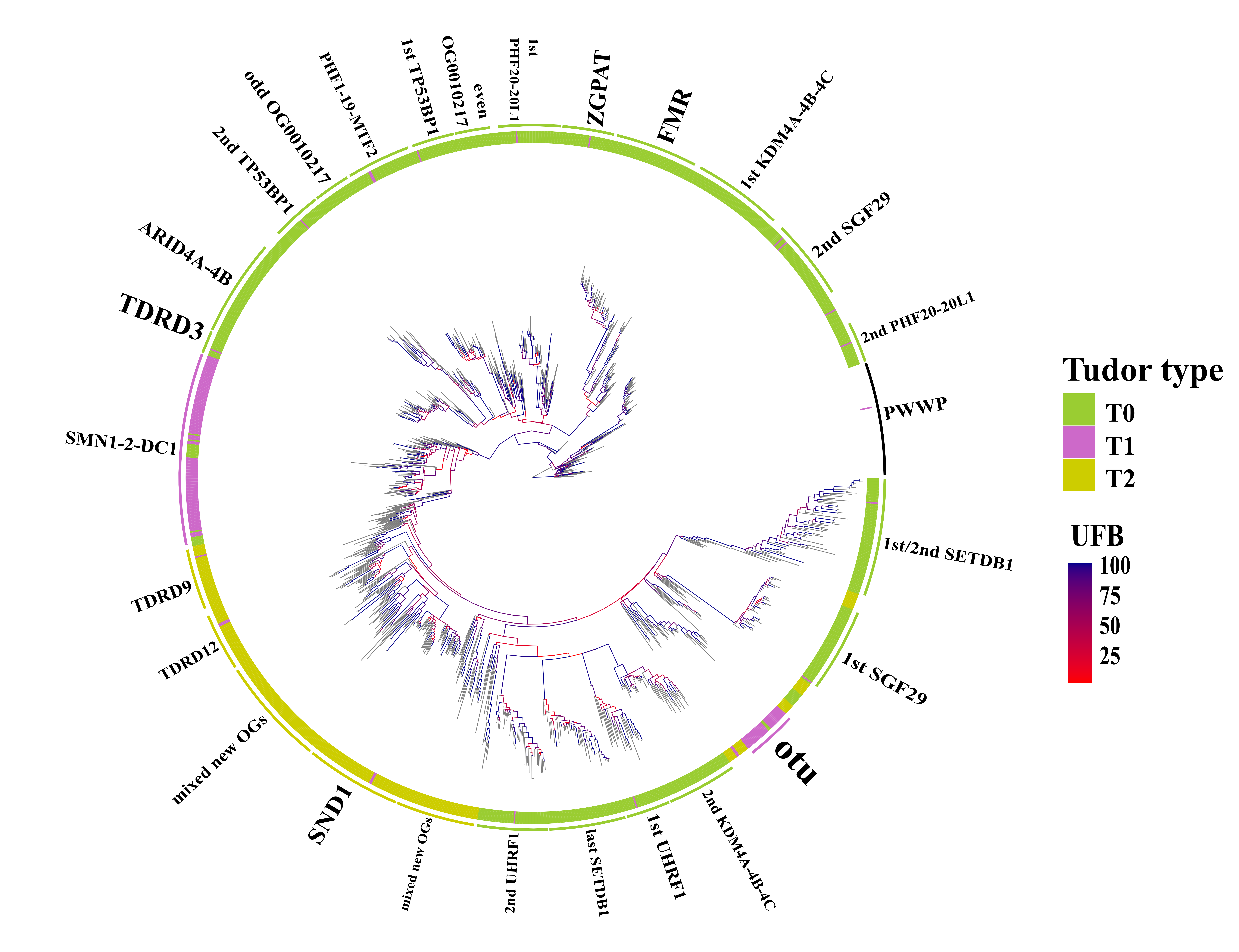

Supplement: evaf051_Supplementary_Data [file evaf051_supplementary_data.zip › Supplementary_Figure_6.png]

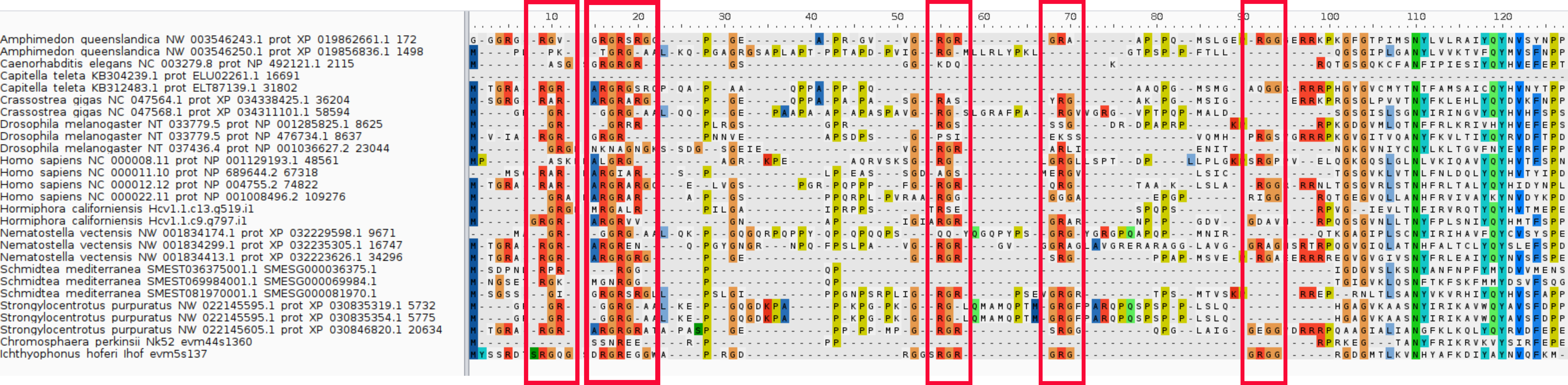

Supplement: evaf051_Supplementary_Data [file evaf051_supplementary_data.zip › Supplementary_Figure_7.png]

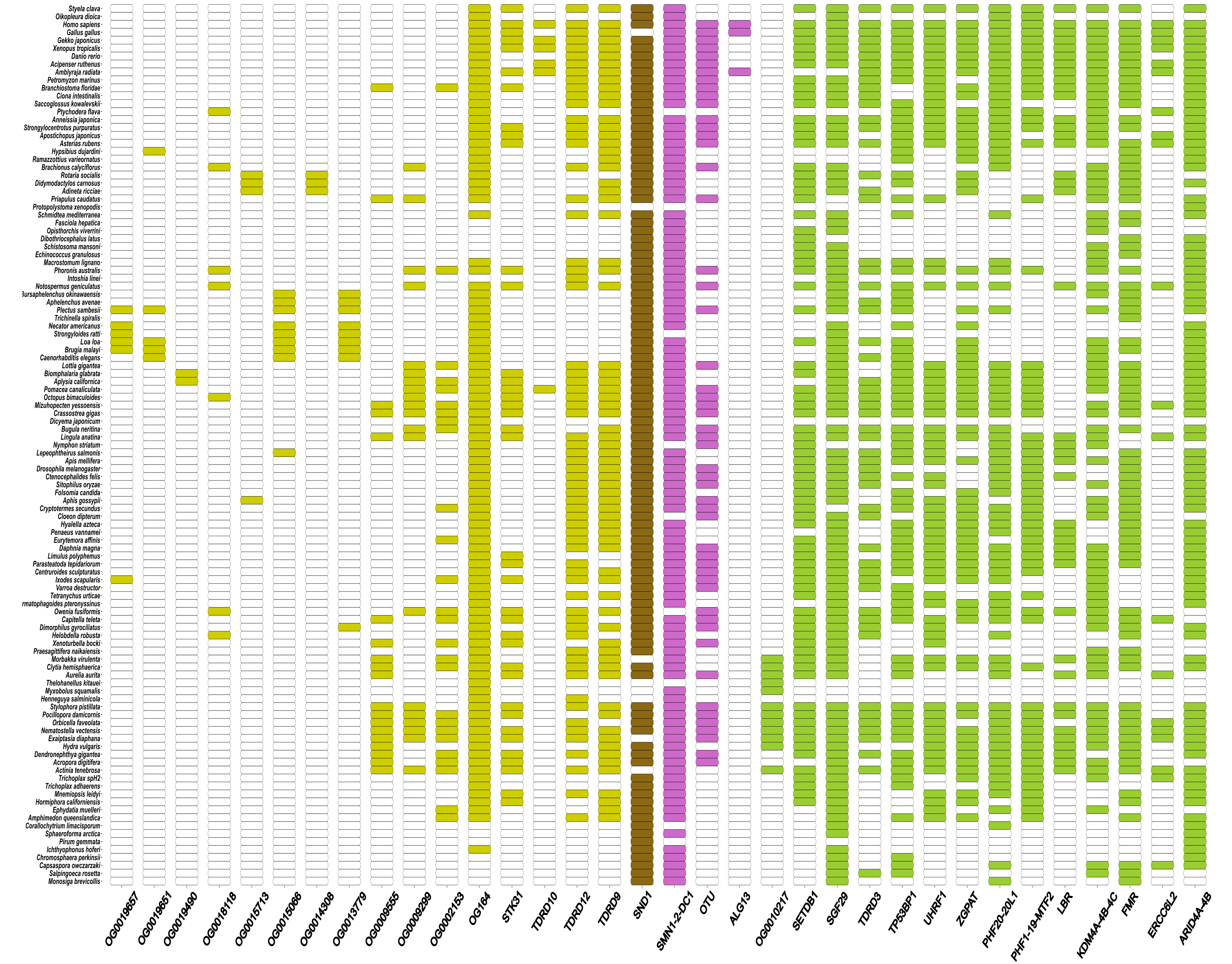

Supplement: evaf051_Supplementary_Data [file evaf051_supplementary_data.zip › Supplementary_Figure_8.png]

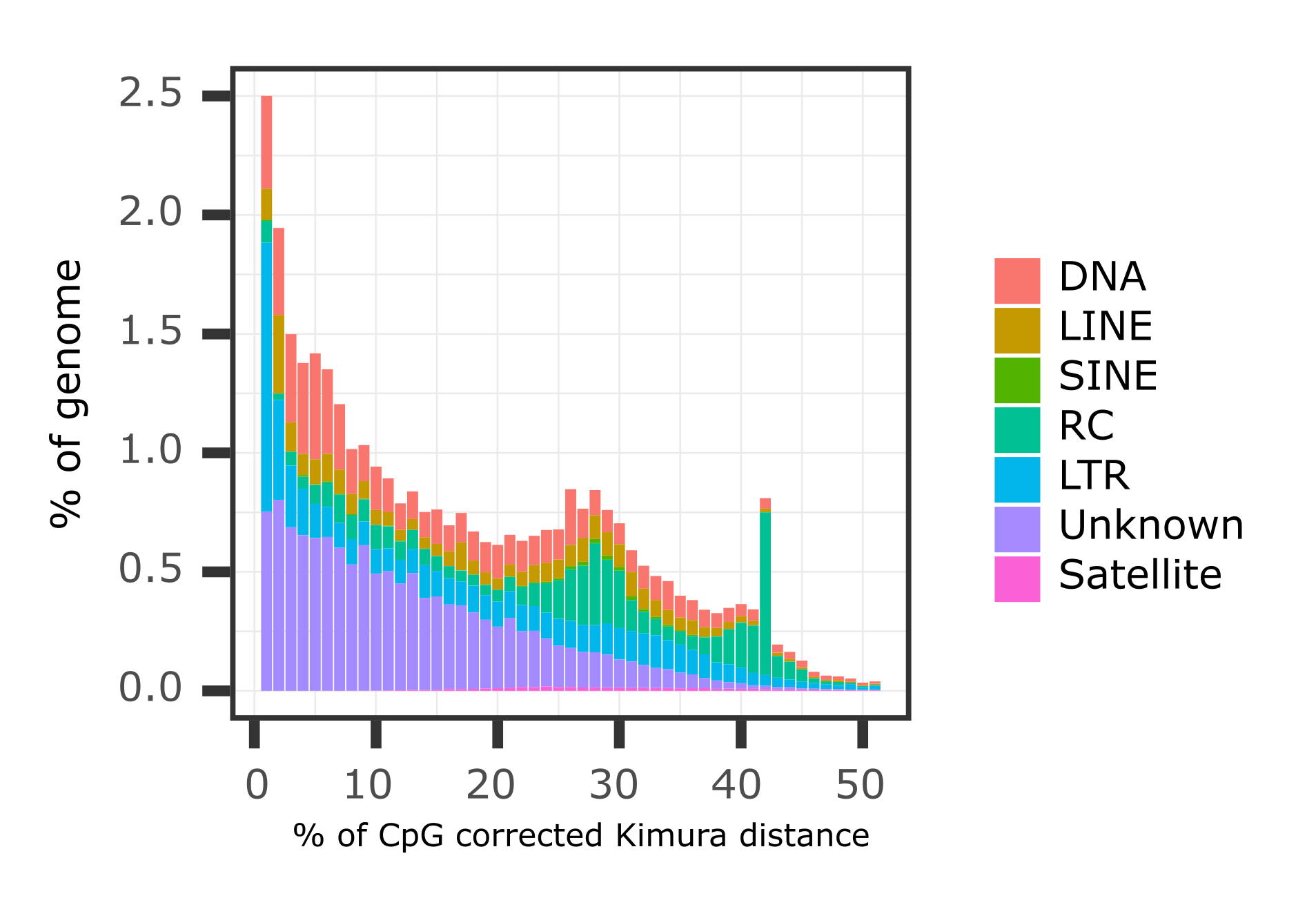

Supplement: evaf051_Supplementary_Data [file evaf051_supplementary_data.zip › Supplementary_Figure_9.png]
